# Supplementary material for: Trend, burden and determinants of undiagnosed hypertension in the Horn of Africa: A systematic review and meta-analysis
Source: PLoS One. 2024 Aug 23;19(8):e0303940. doi: 10.1371/journal.pone.0303940 (PMC11343413; doi:10.1371/journal.pone.0303940)
Supplement: S1 File — (DOCX) [file pone.0303940.s003.docx]

**Supporting file 1: A search strategy for the study of undiagnosed hypertension in the Horn of Africa**

| **Entry terms** | **Combination** | **# Of article** | **Last date of searching** | **Electronic Data bases**  **& webs** |
| --- | --- | --- | --- | --- |
| undiagnosed hypertension, hypertension, high blood pressure, Ethiopia, Eritrea, Sudan, Kenya, Uganda, Djibouti, Somali, South Sudan | Undiagnosed hypertension OR hypertension OR high blood pressure AND Ethiopia OR Djibouti OR Eritrea OR Somali OR Uganda OR Sudan OR South Sudan OR Kenya | 87,779 | January 23, 2023 | Medline/PubMed |
|  | Undiagnosed hypertension OR hypertension OR high blood pressure AND Ethiopia OR Djibouti OR Eritrea OR Somali OR Uganda OR Sudan OR South Sudan OR Kenya | 134,371 | January 28, 2023 | EMBASE |
|  |  | 324 | February , 20, 2023 | Google |
|  |  |  |  |  |
|  |  | 25,300 | February 30, 2023 | Google Scholar |
