# Supplementary material for: Trend, burden and determinants of undiagnosed hypertension in the Horn of Africa: A systematic review and meta-analysis
Source: PLoS One. 2024 Aug 23;19(8):e0303940. doi: 10.1371/journal.pone.0303940 (PMC11343413; doi:10.1371/journal.pone.0303940)
Supplement: S2 File — (DOCX) [file pone.0303940.s004.docx]

**Supportive figure-1: sensitivity analysis of un-diagnosed hypertension in the Horn of Africa, 2023.**
