# Supplementary material for: Trend, burden and determinants of undiagnosed hypertension in the Horn of Africa: A systematic review and meta-analysis
Source: PLoS One. 2024 Aug 23;19(8):e0303940. doi: 10.1371/journal.pone.0303940 (PMC11343413; doi:10.1371/journal.pone.0303940)
Supplement: S3 File — (DOCX) [file pone.0303940.s005.docx]

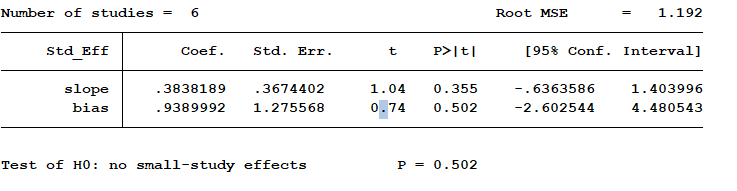

**Supporting figure-2 Effect of age 35-44 years on undiagnosed hypertension in Ethiopia**


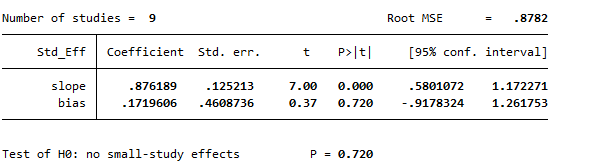

**Supporting figure-3 effect of age >=45 years on undiagnosed hypertension in Ethiopia, 2023**


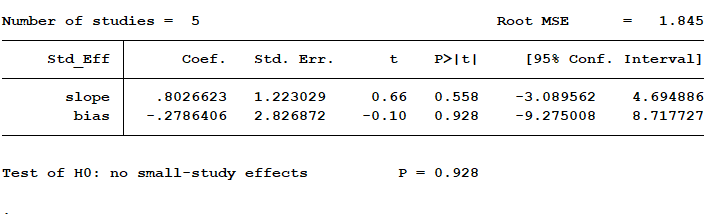

**Supporting figure-4 effect of low consumption of vegetables on undiagnosed hypertension in Ethiopia 2023.**


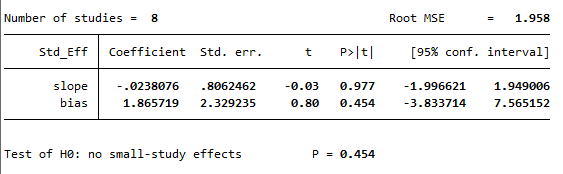


**Supporting figure-5 effect of male on undiagnosed hypertension in Ethiopia, 2023.**


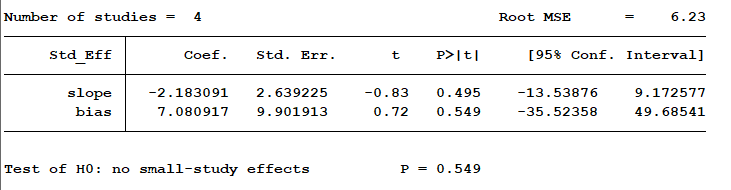

**Supporting figure-6 effects of normal BMI on undiagnosed hypertension in Ethiopia,2023.**


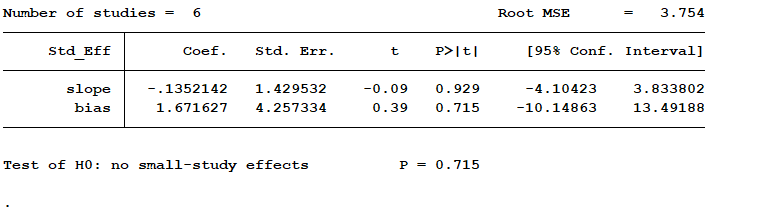

**Supporting figure-7 effects of smoking on undiagnosed hypertension in Ethiopia,2023**


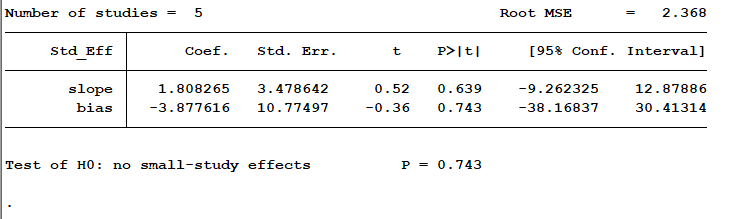

**Supporting figure-8 effect of alcohol on undiagnosed hypertension in Ethiopia,2023**


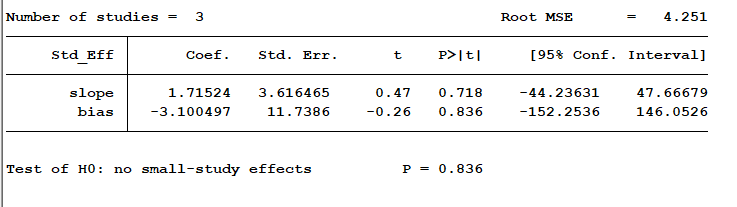

**Supporting figure-9 effect of chat chewing on undiagnosed hypertension in Ethiopia, 2023**

**Supporting figure-10 effect of time to reach health facilities on undiagnosed hypertension in Ethiopia, 2023**


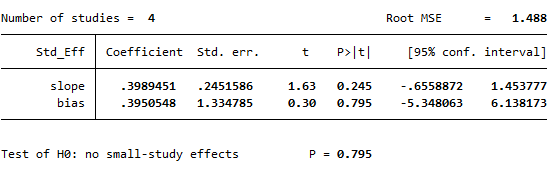

**Supporting figure-11 effects of the high level of triglyceride undiagnosed hypertension in Ethiopia, 2023**


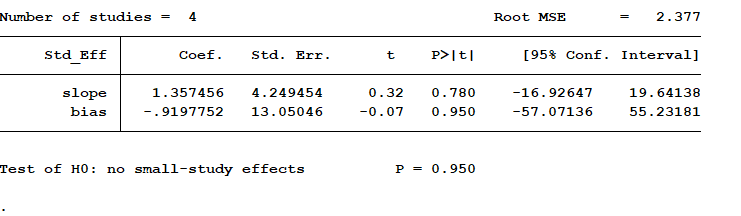


** Supporting figure-12 effect of low knowledge about hypertension on undiagnosed hypertension in Ethiopia, 2023**


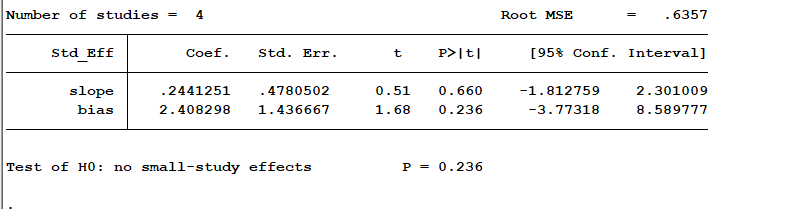

**Supporting figure-13 effect of low health seeking on undiagnosed hypertension in Ethiopia, 2023**


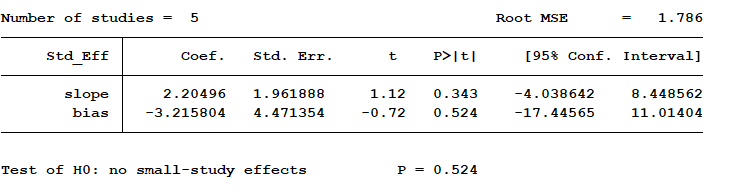

**Supporting figure-14 effect of no regular exercise on undiagnosed hypertension in Ethiopia, 2023.**


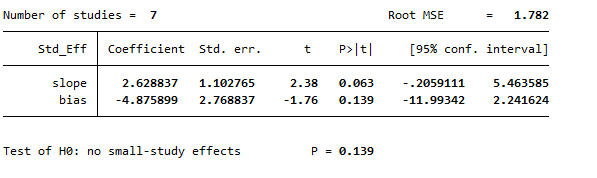

**Supporting figure-15 effect of overweight on undiagnosed hypertension in Ethiopia, 2023.**
