# Supplementary material for: Trend, burden and determinants of undiagnosed hypertension in the Horn of Africa: A systematic review and meta-analysis
Source: PLoS One. 2024 Aug 23;19(8):e0303940. doi: 10.1371/journal.pone.0303940 (PMC11343413; doi:10.1371/journal.pone.0303940)
Supplement: Attachment — Submitted filename: PONE-D-24-00667.pdf [file pone.0303940.s007.pdf]

PLOS ONE

Trend, burden and determinants of undiagnosed hypertension in the horn of Africa: A systematic review and meta-analysis.

--Manuscript Draft--

**Manuscript Number:**	PONE-D-24-00667
**Article Type:**	Research Article
**Full Title:**	Trend, burden and determinants of undiagnosed hypertension in the horn of Africa: A systematic review and meta-analysis.
**Short Title:**	undiagnosed hypertension in the horn of Africa
**Corresponding Author:**	muluken agimas chanie, MPH<br>University of Gondar College of Medicine and Health Sciences<br>Gondar, ETHIOPIA
**Keywords:**	Keyword: undiagnosed hypertension, Ethiopia, meta-analysis, systematic review
**Abstract:**	Abstract<br><br>Background: Hypertension is a silent killer disease that rarely shows signs and symptoms. Because of this nature, most people do not seek medical care early. The global report revealed that half of the world's population lives with undiagnosed hypertension. Among the people who are living with hypertension, half of them are unaware of their status. In the Horn of Africa, hypertension is becoming a major public health agenda. But there is no systematic review and meta-analysis to assess the trend, prevalence, and determinants of undiagnosed hypertension to provide conclusive and tangible evidence about the problem.<br>Objective: To assess the trend, burden, and determinates of undiagnosed hypertension in the Horn of Africa.<br>Method: Published and unpublished papers were searched on Google, Google Scholar, PubMed/Medline, EMBASE, SCOPUS, and the published articles' reference list. The searching mechanism was established using Medical Subject Heading (MeSH) terms by combining the key terms of the title. To assure the quality of the included articles, Joana Brigg's Institute critical appraisal checklist was used, and those articles with a total quality score greater than 50% were considered to have a low risk of bias. A sensitivity test was conducted to evaluate the heterogeneity of the studies. The visual funnel plot test and Egger's and Begg's statistics in the random effect model were done to evaluate the publication bias and small study effect. The I2 statistics were also used to quantify the amount of heterogeneity between the included studies.<br>Result: A total of 43 studies were included for analysis, and the pooled prevalence of undiagnosed hypertension in the Horn of Africa was 17% (95% CI: 15–20). Distance of health facilities takes <35 minute (OR=0.52, 95%CI: 0.35-0.79), no regular exercise (OR=2.26, 95% CI: 1.54-3.32), age >=45 years (OR =2.51, 95% CI: 1.66-3.8), age 35-44 years (OR =1.88, 95% CI: 1.5-2.37), male (OR =1.72, 95% CI: 1.34–2.2), poor knowledge (OR=3.29, 95%CI: 2.39,4.53), normal BMI (OR=3.84, 95% CI: 2.96-4.98), Overweight (OR=1.97, 95% CI: 2.96-4.98), poor health seeking (OR= 2.79, 95%CI: 2.01-3.86), low vegetable consumers (OR= 1.99, 95%CI:1.36-2.91), smoking (OR= 1.47, 95%CI: 1.13-1.93), high triglyceride (OR=1.83, 95%CI:1.33-2.52), chat chewing (OR=2.18, 95%CI: 1.54-3.09), and alcohol drinking (OR=1.75, 95%CI: 1.32-2.33) were the determinats of undiagnosed hypertension.<br>Conclusion and recommendation: The pooled prevalence of undiagnosed hypertension was low in the Horn of Africa. Distance takes <35 min was the protective factors for undiagnosed hypertension. Whereas no regular exercise, age >=45 years, age 35-44 years, male, poor knowledge, normal BMI, overweight, poor health, low vegetable consumption, smokers, high triglyceride, chat chewing, and alcohol drinking were the risk factors of undiagnosed hypertension. Therefore, health professionals and higher officials should promote a healthy lifestyle, encourage early health-seeking behaviour, and expand screening programmes.
**Order of Authors:**	muluken agimas chanie, MPH
	nebiyu Mekonen

Powered by Editorial Manager® and ProduXion Manager® from Aries Systems Corporation

	Fantu Mamo
	Moges Tadesse Abebe
	Tilahun Yemanu
	Meron Asmamaw
**Additional Information:**	
**Question**	**Response**
**Financial Disclosure**<br><br>Enter a financial disclosure statement that describes the sources of funding for the work included in this submission. Review the submission guidelines for detailed requirements. View published research articles from *PLOS ONE* for specific examples.<br><br>This statement is required for submission and **will appear in the published article** if the submission is accepted. Please make sure it is accurate.<br><br>**Funded studies**<br>Enter a statement with the following details:<br>• Initials of the authors who received each award<br>• Grant numbers awarded to each author<br>• The full name of each funder<br>• URL of each funder website<br>• Did the sponsors or funders play any role in the study design, data collection and analysis, decision to publish, or preparation of the manuscript?<br><br>Did you receive funding for this work?	The author(s) received no specific funding for this work.
**Competing Interests**<br><br>Use the instructions below to enter a competing interest statement for this submission. On behalf of all authors, disclose any competing interests that could be perceived to bias this work—acknowledging all financial support and any other relevant financial or non-financial competing interests.	The authors have declared that no competing interests exist.

This statement is required for submission and **will appear in the published article** if the submission is accepted. Please make sure it is accurate and that any funding sources listed in your Funding Information later in the submission form are also declared in your Financial Disclosure statement.<br><br>View published research articles from *PLOS ONE* for specific examples.<br><br>**NO authors have competing interests**<br><br>Enter: *The authors have declared that no competing interests exist.*<br><br>**Authors with competing interests**<br><br>Enter competing interest details beginning with this statement:<br><br>*I have read the journal's policy and the authors of this manuscript have the following competing interests: [insert competing interests here]*<br><br>* typeset	
**Ethics Statement**<br><br>Enter an ethics statement for this submission. This statement is required if the study involved:<br><br>• Human participants<br>• Human specimens or tissue<br>• Vertebrate animals or cephalopods<br>• Vertebrate embryos or tissues<br>• Field research<br><br>Write "N/A" if the submission does not require an ethics statement.<br><br>General guidance is provided below. Consult the submission guidelines for detailed instructions. **Make sure that all information entered here is included in the Methods section of the manuscript.**	N/A

Powered by Editorial Manager® and ProduXion Manager® from Aries Systems Corporation

**Format for specific study types**<br><br>**Human Subject Research (involving human participants and/or tissue)**<br>• Give the name of the institutional review board or ethics committee that approved the study<br>• Include the approval number and/or a statement indicating approval of this research<br>• Indicate the form of consent obtained (written/oral) or the reason that consent was not obtained (e.g. the data were analyzed anonymously)<br><br>**Animal Research (involving vertebrate animals, embryos or tissues)**<br>• Provide the name of the Institutional Animal Care and Use Committee (IACUC) or other relevant ethics board that reviewed the study protocol, and indicate whether they approved this research or granted a formal waiver of ethical approval<br>• Include an approval number if one was obtained<br>• If the study involved *non-human primates*, add *additional details* about animal welfare and steps taken to ameliorate suffering<br>• If anesthesia, euthanasia, or any kind of animal sacrifice is part of the study, include briefly which substances and/or methods were applied<br><br>**Field Research**<br><br>Include the following details if this study involves the collection of plant, animal, or other materials from a natural setting:<br>• Field permit number<br>• Name of the institution or relevant body that granted permission	
**Data Availability**<br><br>Authors are required to make all data underlying the findings described fully available, without restriction, and from the time of publication. PLOS allows rare exceptions to address legal and ethical concerns. See the PLOS Data Policy and FAQ for detailed information.	Yes - all data are fully available without restriction

A Data Availability Statement describing where the data can be found is required at submission. Your answers to this question constitute the Data Availability Statement and **will be published in the article**, if accepted.<br><br>**Important:** Stating 'data available on request from the author' is not sufficient. If your data are only available upon request, select 'No' for the first question and explain your exceptional situation in the text box.<br><br>Do the authors confirm that all data underlying the findings described in their manuscript are fully available without restriction?	
**Describe where the data may be found in full sentences. If you are copying our sample text, replace any instances of XXX with the appropriate details.**<br><br>• If the data are **held or will be held in a public repository**, include URLs, accession numbers or DOIs. If this information will only be available after acceptance, indicate this by ticking the box below. For example: *All XXX files are available from the XXX database (accession number(s) XXX, XXX.).*<br>• If the data are all contained **within the manuscript and/or Supporting Information files**, enter the following: *All relevant data are within the manuscript and its Supporting Information files.*<br>• If neither of these applies but you are able to provide **details of access elsewhere**, with or without limitations, please do so. For example:<br><br>*Data cannot be shared publicly because of [XXX]. Data are available from the XXX Institutional Data Access / Ethics Committee (contact via XXX) for researchers who meet the criteria for access to confidential data.*<br><br>*The data underlying the results presented in the study are available*	All relevant data are within the manuscript and its Supporting Information files.

Powered by Editorial Manager® and ProduXion Manager® from Aries Systems Corporation

from (include the name of the third party and contact information or URL).<br>• This text is appropriate if the data are owned by a third party and authors do not have permission to share the data.<br><br>* typeset	
Additional data availability information:	

Powered by Editorial Manager® and ProduXion Manager® from Aries Systems Corporation

Trend, burden and determinants of undiagnosed hypertension in the horn of Africa: A systematic review and meta-analysis.

Muluken Chanie Agimas*[1], Nebiyu Mekonen[1], Fantu Mamo[1], Moges Tadesse Abebe[2] Tilahun Yemanu[1], Meron Asmamaw[1].

[1] Department of Epidemiology and Biostatistics, institute of public health, college of medicine and health science, university of Gondar, Gondar, Ethiopia.

[2]Department of Nursing, College of Health Science, Debark University, Debark, Ethiopia

Authors address:

Muluken Chanie Agimas (MC): mulukensrc12@gmail.com

Nebiyu Mekonen (NM): nebiyumek12@gmail.com

Fantu mamo(FM): fantuma3@gmail.com

Moges Tadesse Abebe (MTA): moges7045@gmail.com

Tilahun Yemanu(TY): yemanu.tilahun@gmail.com

Meron Asmamaw(MA): merryalem101@gmail.com

Corresponding Author

MC: mulukensrc12@gmail.com

Abstract

Background: Hypertension is a silent killer disease that rarely shows signs and symptoms. Because of this nature, most people do not seek medical care early. The global report revealed that half of the world's population lives with undiagnosed hypertension. Among the people who are living with hypertension, half of them are unaware of their status. In the Horn of Africa, hypertension is becoming a major public health agenda. But there is no systematic review and meta-analysis to assess the trend, prevalence, and determinants of undiagnosed hypertension to provide conclusive and tangible evidence about the problem.

Objective: To assess the trend, burden, and determinates of undiagnosed hypertension in the Horn of Africa.

Method: Published and unpublished papers were searched on Google, Google Scholar, PubMed/Medline, EMBASE, SCOPUS, and the published articles' reference list. The searching mechanism was established using Medical Subject Heading (MeSH) terms by combining the key terms of the title. To assure the quality of the included articles, Joana Brigg’s Institute critical appraisal checklist was used, and those articles with a total quality score greater than 50% were considered to have a low risk of bias. A sensitivity test was conducted to evaluate the heterogeneity of the studies. The visual funnel plot test and Egger’s and Begg’s statistics in the random effect model were done to evaluate the publication bias and small study effect. The I^2 statistics were also used to quantify the amount of heterogeneity between the included studies.

Result: A total of 43 studies were included for analysis, and the pooled prevalence of undiagnosed hypertension in the Horn of Africa was 17% (95% CI: 15–20). Distance of health facilities takes <35 minute (OR=0.52, 95%CI: 0.35-0.79), no regular exercise (OR=2.26, 95% CI: 1.54-3.32), age >=45 years (OR =2.51, 95% CI: 1.66-3.8), age 35-44 years (OR =1.88, 95% CI: 1.5-2.37), male (OR =1.72, 95% CI: 1.34–2.2), poor knowledge (OR=3.29, 95%CI: 2.39,4.53), normal BMI (OR=3.84, 95% CI: 2.96-4.98), Overweight (OR=1.97, 95% CI: 2.96-4.98), poor health seeking (OR= 2.79, 95%CI: 2.01-3.86), low vegetable consumers (OR= 1.99, 95%CI:1.36-2.91), smoking (OR= 1.47, 95%CI: 1.13-1.93), high triglyceride (OR=1.83, 95%CI:1.33-2.52), chat chewing (OR=2.18, 95%CI: 1.54-3.09), and alcohol drinking (OR=1.75, 95%CI: 1.32-2.33) were the determinats of undiagnosed hypertension.

Conclusion and recommendation: The pooled prevalence of undiagnosed hypertension was low in the Horn of Africa. Distance takes <35 min was the protective factors for undiagnosed hypertension. Whereas no regular exercise, age >=45 years, age 35-44 years, male, poor knowledge, normal BMI, overweight, poor health, low vegetable consumption, smokers, high triglyceride, chat chewing, and alcohol drinking were the risk factors of undiagnosed hypertension. Therefore, health professionals and higher officials should promote a healthy lifestyle, encourage early health-seeking behaviour, and expand screening programmes.

Keyword: undiagnosed hypertension, Ethiopia, meta-analysis, systematic review

Introduction

Hypertension can be defined by two or more repeated measurements of systolic blood pressure ≥ 130mm Hg or ≥80 for diastolic pressure (1). Hypertension is a silent killer disease that rarely shows signs and symptoms. Because of this nature of the disease, most people do not seek medical care and go to health institutions when the disease becomes more advanced or complicated (2). Unmanaged hypertension leads to stroke, premature death, heart disease, chronic kidney disease, and other causes of mortality (3). Approximately one billion people have developed hypertension on the globe and if essential intervention is not given, by 2025 hypertension will be increased to 1.5 billion cases (4). The global report revealed that half of the world's population lives with undiagnosed hypertension (5). Among the people who are living with hypertension, half of them are unaware of whether they are living with hypertension or not (6). Increased blood pressure can cause 9 million ischaemic heart diseases and 3.5 million strokes (7).

Of the global hypertension cases, Africa contributes about 46% (8). Furthermore, the specific death rate of hypertension in Africa accounts for 25% of the total death (9). Even though the burden of hypertension is high, most African countries can not afford the health service cost because there are other prioritized health conditions and an inadequate supply of drugs and other medical equipment (10). Nations around the world, including the horn of Africa, have been working to meet the target of sustainable development goal target 3.4 of reducing the premature mortality rate of non-communicable disease by one-third through early prevention and control in 2030 (11).

Undiagnosed hypertension in the horn of Africa is becoming a major public health agenda. Evidence shows that hypertension has become a hidden epidemic (12, 13). Undiagnosed hypertension can be attributed/associated with smoking, an unhealthy diet (for example, high intake of salt, fat, and refined sugar stress), lack of regular physical activity, body mass index of greater than 25, older age, and alcohol drinking/unhealthy lifestyle (14, 15). The horn African countries has been working to reduce the health and economic impact of hypertension, but still, the magnitude of the problem is rising. Furthermore, there is no updated information about the burden, trend and detreminats of undiagnosed hypertension in the horn of Africa. Thus, the aim of this systematic review and meta-analysis was to try to determine the trend, prevalence anddeterminats of undiagnosed hypertension in the horn of Africa.

Methodology

Design

The Preferred Reporting Items for Systematic Reviews and Meta-Analysis Protocols (PRISMA-P 2015 Guidelines) were used to design the protocol (16).

Searcing strategy

All the important published and unpublished papers were searched on, Google, Google Scholar, PubMed/Medline, EMBASE, SCOPUS databases, and the published article's reference list from January 23 to February 30/2023. The searching mechanism was established using Medical Subject Heading (MeSH) terms by combining the key terms of the title. Again, reviewing the published articles reference list was used to identify those papers which are missing on Google, Google Scholar, PubMed/Medline and EMBASE.

Content experts were advised to obtain more papers or to reduce the risk of missing. Six authors (Muluken chanie Agimas, Meron Asmamaw, Moges Tadesse Abebe, Nebyu Mekonnen, Fantu Mamo, Tilahun Yemanu) searched the articles independently, and cross cheeking their results.This systematic review and meta analysis was conducted by extraction of studies in the context of prevalence of undiagnosed hypertention using the following key entry terms. "Undiagnosed hypertension OR hypertension OR high blood pressure AND Ethiopia OR Djibouti OR Eritrea OR Somali OR Uganda OR Sudan OR South Sudan OR Kenya" **(Table-1)**.

Table-1: a searching strategy for the study undiagnosed hypertention in the horn of Africa.

Study selection and quality appraisal

The retrieved articles were imported into EndNote X8 software. The duplicated articles were removed after all articles were imported into one database with endnote X8. The title and abstracts of each articles were screened by paired authors (MC and NM). The discrepancy among the paired authors was solved by discussion and other review teams (MA, FM, DA, TY and MTA). Articles with full text were considered for systematic and meta-analysis. To assure the quality of the retrieved and included articles, Joana Brigg's Institute critical appraisal checklist for simple prevalence (17) and analytical cross-sectional studies (18) having nine and eight checklist items respectively were considered. Articles with a total quality score of more than 50% were labeled as paper-qualified articles was a low risk of bias (18). During the quality appraisal, these discrepancy was solved by discussion.

Eligibly criteria

Inclusion criteria

Articles/studies met the following criteria were include in the systematic and meta analysis:

- Studies have reported the prevalence and/or associated factors of undiagnosed hypertension in the horn of Africa.
- All cross-sectional, prospective cohort studies, case control studies and intervetional studies were considered.
- Those articles published in English language and published at any period.

Exclusion Criteria

- Articles not report the outcome of interest were excluded.

Outcomes of the study

The primary outcome of the intended systematic and meta-analysis was the prevalence of undiagnosed hypertension and associated factors in the horn of Africa.

Operational definition

Undiagnosed hypertention: was dened as Systolic blood preasure 140 mmHg and above, and/or diastolic blood prasure 90 mmHg and above, without previous history or anti-hypertensive treatment during survey diagnosis (19).

Data extraction

For the purpose of extracting the data, the JBI tool was adopted. The six authors extracted the data independently. The discrepancy was solved by discussion. Name of the first author, publication year, study area/region, study design, tool, quality of the articles, number of samples, number of undiagnosed hypertension, proportions, standard error of the proportion, odds ratio for each independent variable, confidence interval of the odds ratio and standard error of each odds ratio were extracted from each studies using Microsoft excel spreadsheet.

Data analysis

Data from the eligible articles were entered into Microsoft Excel and then exported to Stata-14 for further data analysis. The I^2 statistics or index of heterogeneity was used to quantify the amount of heterogeneity between the included studies (20). Values of I^2 25%, 50%, and 75% were considered as low, medium, and high heterogeneity respectively (21). The significance of the heterogeneity between the studies was evaluated by a p-value of I^2 less than 0.05. Sensitivity and analysis and subgroup anayslis using the study setting and regions was conducted to evaluate and manage the heterogeneity. The visual funnel plot test (visual method) and Egger's statistics (objective/statistical technique) in the random effect model were done to evaluate the publication bias/small study effect. The effect size was reported by OR with its 95% confidence level to estimate the statistical association between undiagnosed hypertension and associated factors. Prevalence of undiagnosed hypertension and factors was reported by both the text and the forest plot.

Results

Out of 247,774 searched articles, 243,987 were excluded because of duplicated articles. About 2519 records were screened and 264 of them were assessed for elligiblity. Finaly 43 potential studies have been included for systematic review and meta analysis as summarized in the PRISMA flow diagram **(Figure-1).**

Figure-1 PRISMA flow diagram of study selection for the trend, prevalence and determinants of undiagnosed hypertension in the horn of Africa, 2023.

Characteristics of included studies

All included studies were conducted using a cross conditional study design. Of the total included studies, the majority 28 (65.1%) were conducted in the urban setting. The total number of participants included/used for analysis was 64,156 participants and the minimum and maximum sample size used in the studies was 170 (22) and 17,777 (23) respectively **(Table 2).**

Table 2. Characteristics of the included studies in the horn of Africa.

The pooled prevalence of undiagnosed hypertension in the horn of Africa.

In the random effects model, the pooled prevalence of undiagnosed hypertension in the horn of Afria was 17% (95% CI, 15%–20%) and the heterogeneity among the studies was statisticaly significant (I^2 = 98.42%, Pvalue < 0.001) **(Figure-2).**

Figure-2: The pooled prevalence of undiagnosed hypertension in the horn of Africa, 2023.

Assessment of publication bias

Egger'sstatistical test evidenced that was no publication bias among the included studies (β = 7.947, P-value = 0.158) **(Figure-3).**

Figure-3: funnel plot of undiagnosed hypertension in the horn of Africa

Handling heterogenity

In the random effect model of pooled prevalence, a statistical significant heterogeneity was detected. To solve this problem, heterogeneity sensitivity analysis, and subgroup analysis were performed.

Sensitivity analysis

To manage the influence of a single study in meta-analysis estimation (to assess the hetroginiety) the random effects model and sensitivity analysis was conducted and there was no study that excessively influence the overall pooled prevalence of undiagnosed hypertension (**S-1**).

Subgroup analysis by country

In the subgroup analysis by country, the highest prevalence of undiagnosed hypertension was observed in Sudan 28% (95%CI: 16%-40%) and the lowest prevalence of undiagnosed hypertension was reported in Ertirea, 13% (95%CI: 11%-14%) **(Figure-4).**

Figure-4: The pooled prevalence of undiagnosed hypertension in the horn of Africa.by country, 2023

Subgroup analysis by study setting

Subgroup analysis was also carried out by the study setting as a source of heterogeneity. The highest prevalence of undiagnosed hypertension by study setting was observed in rural areas 27% (95% CI: 14%-40%) **(Figure-5).**

Figure-5: The pooled prevalence of undiagnosed hypertension in the horn of Africa by study setting, 2023.

Trends of undiagnosed hypertension in the horn of Africa

The trend of undiagnosed hypertension in the horn of Africa was increased across a time from 13% in 2006 to 20% in 2023 **(Figure-6).**

Figure-6: the trend of undiagnosed hypertension in the horn of Africa

Factors associated with undiagnosed hypertension

As briefly described in **Table-3**, sex, age, BMI, chat chewing, alcohol consumption, regular exercise, knowledge about hypertension, health-seeking behavior, vegetable consumption, level of triglyceride, and time to reach the health facilities were statistically significant factors for undiagnosed hypertension in horn of Africa. From the random effect model estimate, the pooled odds of undiagnosed hypertension among males were 1.81 times higher than females (OR =1.81, 95% CI: 1.42–2.3); with I^2 =72.5%, P-value =0.001. Egger's test indicates that there was no small study effect (P-value =0.454) and in random effects model reported no single study that excessively influences the effect size of this factor **(Table-3, S-2).**

Another significant factor for undiagnosed hypertension was age and thus age >=45years and 35-44 years was 2.49 (OR = 2.49, 95% CI: 2.09-2.97), (I^2 =0%, P-value=0.72), 1.88 (OR = 1.88, 95% CI: 1.5-2.37, I^2 =22.5%, P-value=0.266) times higher risk for undiagnosed hypertension than 18-34 years respectively. The egger's test indicates that there was no small study effect in age >=45 years (P-value =0.444) and 35-44 years (P=0.502). This means, in the random effects model there was no single study that excessively influences the effect size of this factor**(Table-3, S-2).**

The pooled effect size of undiagnosed hypertension among those who had no regular exercise was 2.2 times more likely than the counterpart (OR=2.26, 95% CI: 1.54-3.32, I^2 =64.3%, P-value =0.023). Egger's test indicates that there was no small study effect (P-

value = 0.524) and in the random effect model there was no single study that excessively influenced the effect size of this factor **(Table-3, S-2).**

The pooled odds of undiagnosed hypertension in < 35 minutes to reach the health facilities was reduced by 48% than >=35 minutes to reach the health facilities (OR=0.52, 95% CI: 0.35-0.79 with I^2=88.6%, P-value =0.003). Egger's test indicates that there was no small study effect (P-value=0.245) and in the random effects model there was no single study that excessively influenced the effect size of this factor **(Table-3, S-2).**

Regarding knowledge of hypertension, adults who had poor knowledge about hypertension were 3.29 times more likely to have undiagnosed hypertension than good knowledge about hypertension (OR=3.29, CI: 2.39,4.53) with I^2=43.7%, P-value =0.137. Egger's test indicates that there was no small study effect (P-value =0.95) and thus there was no single study that excessively influences the effect size of this factor **(Table-3, S-2).**

Again, the odds of undiagnosed hypertension among normal BMI and overweight were 3.84 (OR=384, 95% CI: 2.96-4.98, I^2=96.1%, P-value=<0.001) and 2.11 (OR=2.11, CI: 1.57-2.83, I^2=76.7%, P-value=<0.001) times higher than underweight. Egger's test indicates that therewas no small study effect for normal BMI (P-value =0.549), for overweight (P-value=0.139), and thus there was no single study that excessively influences the effect size of this factor **(Table-3, S-2).**

Having poor health-seeking behavior was 2.79 times more likely to have undiagnosed hypertension than good health-seeking behavior (OR=2.79, CI: 2.01-3.86 with I^2=0%, P-value =0.646). Egger's test also indicates that there was no small study effect (P-value

=0.236) and thus there was no single study that excessively influences the pooled estimate of this factor **(Table-3, S-2).**

Another factor that influences undiagnosed hypertension was vegetable consumption. Thus, the odds of undiagnosed hypertension among low vegetable consumers were 1.99 times higher than normal consumers (OR=1.99, 95% CI: 1.36-2.91 with I^2=61%, P-value=0.036). Egger's test indicates that there was no small study effect (P-value =0.928) and thus there was no single study that excessively influences the effect size of this factor **(Table-3, S-2).** Additionally, the odds of undiagnosed hypertension among smokers were 1.47 times higher than nonsmokers (OR= 1.47, 95% CI: 1.13-1.93 with I^2=91.5%, P-value<0.001). Egger's test indicates that there was no small study effect (P-value =0.715) and thus there was no single study that excessively influences the effect size of this factor **(Table-3, S-2).** Having a high level of triglyceride was 1.58 times more likely at risk for undiagnosed hypertension than a normal level of triglyceride (OR=1.58, 95% CI:1.32-1.89 with I^2=35.1%, P-value=0.202). Egger's test showed that there was no small study effect (P-value =0.795) and thus there was no single study that excessively influenced the effect size of this factor **(Table-3, S-2).**

The odds of undiagnosed hypertension among chat chewers were 2.18 times higher than the counterpart (OR=2.18, CI: 1.54-3.09 with I2=89.7%, P-value<0.001). Egger's test indicates that there was no small study effect (P-value=0.836) and thus there was no single study that excessively influences the effect size of this factor **(Table-3, S-2).**

On the other hand, the odds of undiagnosed hypertension among alcohol drinkers were 1.75 times higher than the counterparts (OR=1.75, 95% CI: 1.32-2.33 with I^2=77.2%, P-

value=0.002). Egger's test also showed that there was no small study effect (P-value =0.743) and thus there was no single study that excessively influences the effect size of this factor **(Table-3, S-2).**

Table 3. Summary of factors associated with undiagnosed hypertension in the horn of Africa., 2023

Discussion

In this study, an attempt has been made to assess the trend, burden and determinats of undiagnosed hypertension in the horn of Africa.. Thus the pooled prevalence of undiagnosed hypertension in in the horn of Africa was 17% (95% CI: 15–20) and Distance takes <35 min, no regular exercise >=45 years, 35-44 years, male, good knowledge, normal BMI, overweight, poor health seeking, low vegetable consumers, smokers, high triglyceride, chat chewing and alcohol drinking were the factors associated with undiagnosed hypertension.

The pooled prevalence of undiagnosed hypertention in the current study was lower than a systematic and meta-analysis study conducted in Sub-Saharan Africa (30%), Nigeria (28.9%), India (29.8%), Pakistan (26.34%), and a study in Nepal (25.1%) (14, 64-68). This may be because of the difference in the time of study and the difference in the study setting. The other possible reason may be that most of the studies conducted and included in the current study were among the urban population. This segment of the population has better health-seeking and check-up behavior than the rural population. In the subgroup analysis by study setting and regions, the pooled prevalence of undiagnosed hypertension was higher in rural areas, which was 25% (95 % CI: 12-38). The possible reason for this difference may be because of the rural population has lower educational status, lack of access to health

facilities, and lack of access to the media, and in return, they may have low health-seeking or health check-up behavior (69).

This systematic and meta-analysis also identifies the associated factors of undiagnosed hypertension in the horn of Africa and, thus being male was more likely to have undiagnosed hypertension than females. This finding was supported by a study done in the United States of America (70). The possible reason for this finding may be most of the time, women have the chance of various maternal health services. For example, family planning delivery, antenatal care services, and immunization services provide a good opportunity for understanding their health status (24).

The odds of undiagnosed hypertension in ages 35-44 years and >=45 years was more than in ages 18-34 years. This finding is supported by studies done in Nepal (71), Uganda (72) and Benin (73) This is due to arterial stiffness becoming increased as age increases. In return, it increases blood pressure and parallelly undiagnosed hypertension is more in older ages (71).

Physical exercise was also a significant factor for undiagnosed hypertension and, thus, adults with no regular exercise were at higher risk of undiagnosed hypertension than regular physical exercise. This is because physical activity and/or exercise are shown to delay the development of hypertension (74). The other possible reason might be associated with those individuals who live a sedentary life, such as those who have no exercise are also not seeking medical care.

This systematic review and meta-analysis also revealed that the distance from health facilities to home affects undiagnosed hypertension and thus traveling time <35 minutes

reduces the risk of undiagnosed hypertension. This may be because those who are at nearby health facilities access the health service more easily than those who are far away.

Poor knowledge about hypertension is another risk factor for undiagnosed hypertension. The finding was supported by the studies conducted in Rwanda and Cracow (5, 75). This might be because adults who have low knowledge about hypertension have a low seek of screening for hypertension.

Another associated factor for undiagnosed hypertension in the horn of Africa.was BMI. Normal BMI and overweight increase the risk of undiagnosed hypertension more than underweight individuals. This is supported by studies conducted in sub-Saharan African countries (76) and national study of Burkina Faso (77). This might be because of a 1 kg/m^2 increase in BMI, the odds of hypertension also increased as compared to under weight (risk of undiagnosed hypertension= obese/overweight > normal > underweight) (78). Alternatively, as increased in the BMI by 1 kg/m^2, there is an increment in the risk of undiagnosed hypertension. In this case normal BMI is higher risk of undiagnosed hypertension as compared to underweight (low BMI) by held other factors constant. Again those individuals who have normal BMI may have poor belief that they have a low risk of developing hypertension which makes them less likely to visit a health institutions to measure their blood pressure. Alternatively, their subjective perception of the lower risk of acquiring hypertension may be refrain to measure their blood pressure. Even during the selective/risk group screening, these individuals may be missed.

In this study, adults having low health-seeking behavior were more likely to have undiagnosed hypertension than good health-seeking behavior. This might be because adults

with low health-seeking behavior have less chance to visit health institutions and, as a result, they do not know their hypertension status (79).

This study also showed that chat chewers are more likely to have undiagnosed hypertension. This is supported by systematic and meta-analysis study in Ethiopia (80). This is due to chat (Khat) contains cathinone, cathine, and amphetamine, which elevate the dopamine in the brain (81) and this leads to elevate the blood preasure (82, 83). Another possible reseon might be adults living in unhealthy life are give less attention for early health seeking/screening (84).

In the current study the pooled effect of alcohol drinking was the risk factor for undiagnosed hypertension. This is due to alcohol drinking negatively affect the centeral nerveous system which increase the cardiacoutput and affects the peripheral vascular effect this leads to elevate the blood preasure (85). Another possible reason might be that adults living an unhealthy life style are given less attention to early health-seeking/screening (84).

Another finding in the current study was adults with low consumption of vegetables were more likely at risk for undiagnosed hypertension. This is because vegetables and fruit are very essential food components of a healthy diet that can prevent a variety of cardiovascular diseases, including hypertension, and WHO recommended these food items to enhance the health of the community (86).

Additionally, the current study reported that alcohol drinkers had a higher risk of undiagnosed hypertension than non-alcohol drinkers. The finding is consistent with studies conducted in Tanzania (87) in China (88, 89). This might be because adults with an unhealthy lifestyle, including alcohol drinking are less health-care seeking behavior (84).

Furthermore, the odds of undiagnosed hypertension was higher among adults with high triglyceride level than normal level of triglyceride. This might be because high triglycerides are often a sign of other conditions that increase the risk of heart disease, hypertension, and other chronic diseases and such adults are under comorbidity. As a result, they seek medical care, have frequent contact with health professionals, and know their blood pressure status. The strength of the study was the involvement of many experts (data extraction by 6 researchers) during the searching, screening, appraisal, and data extraction processes, research helped to make the evidence more comprehensive and valid. Furthermore, the data report adheres to guidelines outlined in the PRISMA P-2015 statement protocol. But some studies categorizing the variables in a different way and are difficult to manage were omitted.

Conculusion: The pooled prevalence of undiagnosed hypertension was low in the horn of Africa. Distance takes <35 minute was the protective factors for undiagnosed hypertension whereas no regular exercise >=45 years, 35-44 years, being male, good knowledge, normal BMI, overweight, poor health seeking, low vegetable consumption, smokers, high triglyceride, chat chewing and alcohol drinking were the risk factors of undiagnosed hypertension. Therefore the health professionals and higher officials better to promote healthy life style, early health seeking behavior and expand the screening programs.

Ethical declaration

Ethical approval and consent to participate

Because it was a systematic review and meta-analysis, ethical approval and consent to participate was not necessary.

Consent for publication

Not applicable.

Data availability statement

All relevant data are within the paper and its supporting information files.

Conflict of interest

The author declares no conflict of interest

Source of funding

The authors received no specific funding for this work.

Acknowledgments

The authors acknowledged University of Gondar staffs for their support.

Supporting information

S-1: sensitivity analysis of undiagnosed hypertension in the horn of Africa, 2023

S-2: Effect of each factor on undiagnosed hypertension in the horn of Africa, 2023

Author Contributions

Conceptualization: Muluken Chanie Agimas, Meron Asmamaw, Nebyu Mekonnen, Fantu Mamo

Formal analysis: Muluken Chanie Agimas, Tilahun Yemanu, Moges Tadesse Abebe

Investigation: Muluken Chanie Agimas , Fantu Mamo

Methodology: Muluken Chanie Agimas, Nebyu Mekonnen

Software: Tilahun Yemanu, Muluken Chanie Agimas , Meron Asmamaw,

Supervision: Muluken Chanie Agimas and Meron Asmamaw.

Validation: Fantu Mamo, Muluken chanie Agimas

Visualization: Muluken Chanie Agimas, Nebyu Mekonnen, Moges Tadesse Abebe

Writing – review & editing: Tilahun Yemanu, Muluken Chanie Agimas, meron Asmamaw, Fantu Mamo, Moges Tadesse Abebe

Abbreviation and acronym

BMI….. Body Mass Index

CI…….Confidence Interval

OR……Odds Ratio

PRISMA…….Prefered Reporting Item for Systematic Review and Meta Analysis

WHO…….World Health Organization

Reference

1. Carey RM, Whelton PK, Committee* AAHGW. Prevention, detection, evaluation, and management of high blood pressure in adults: synopsis of the 2017 American College of Cardiology/American Heart Association Hypertension Guideline. Annals of internal medicine. 2018;168(5):351-8.
2. Day W. A global brief on hypertension. Geneva: World Health Organization. 2013.
3. Lawes CM, Vander Hoorn S, Rodgers A. Global burden of blood-pressure-related disease, 2001. The Lancet. 2008;371(9623):1513-8.
4. Chockalingam A. Impact of world hypertension day. Canadian journal of cardiology. 2007;23(7):517-9.
5. Mazimpaka C, Nsanzimana S, Logan J, Binagwaho A, Wong R. Assessing the Magnitude and Risk Factors Associated With Undiagnosed Hypertension in Rural Rwanda. Assessing the Magnitude and Risk Factors Associated With Undiagnosed Hypertension in Rural Rwanda.
6. Gulec S. Early diagnosis saves lives: focus on patients with hypertension. Kidney international supplements. 2013;3(4):332-4.
7. Williams B, Mancia G, Spiering W, Rosei E, Azizi M. ESC/ESH Guidelines for the management of arterial hypertension [2018 ESC/ESH Guidelines for the management of arterial hypertension]. Kardiol Pol. 2019;77(2):71-159.
8. Organization WH. Global Health Observatory (GHO) data: NCD mortality and morbidity. Retrieved August. 2016;23:2016.

9. Organization WH. Global health risks: mortality and burden of disease attributable to selected major risks: World Health Organization; 2009.
10. Palafox B, McKee M, Balabanova D, AlHabib KF, Avezum Jr A, Bahonar A, et al. Wealth and cardiovascular health: a cross-sectional study of wealth-related inequalities in the awareness, treatment and control of hypertension in high-, middle-and low-income countries. International journal for equity in health. 2016;15(1):1-14.
11. Norheim OF, Jha P, Admasu K, Godal T, Hum RJ, Kruk ME, et al. Avoiding 40% of the premature deaths in each country, 2010–30: review of national mortality trends to help quantify the UN Sustainable Development Goal for health. The Lancet. 2015;385(9964):239-52.
12. Undavalli VK, Mp H. Prevalence of undiagnosed hypertension: a public health challenge. Int J Community Med Public Health. 2018;5(4):1366-70.
13. Gerensea H, Teklay H. Trend of hypertension morbidity and mortality in Tigray Region from 2011 to 2015, Tigray, Ethiopia. BMC research notes. 2018;11(1):1-5.
14. Ataklte F, Erqou S, Kaptoge S, Taye B, Echouffo-Tcheugui JB, Kengne AP. Burden of undiagnosed hypertension in sub-saharan Africa: a systematic review and meta-analysis. Hypertension. 2015;65(2):291-8.
15. Mogas SB, Tesfaye T, Zewde B, Tesfaye Y, Kebede A, Tadesse M, et al. Burden of undiagnosed hypertension among adults in urban communities of Southwest Ethiopia. Integrated Blood Pressure Control. 2021;14:69.
16. Moher D, Shamseer L, Clarke M, Ghersi D, Liberati A, Petticrew M, et al. Preferred reporting items for systematic review and meta-analysis protocols (PRISMA-P) 2015 statement. Systematic reviews. 2015;4(1):1-9.
17. Munn Z, Moola S, Lisy K, Riitano D, Tufanaru C. Methodological guidance for systematic reviews of observational epidemiological studies reporting prevalence and cumulative incidence data. International journal of evidence-based healthcare. 2015;13(3):147-53.
18. Fan S-J, Heinrich J, Bloom MS, Zhao T-Y, Shi T-X, Feng W-R, et al. Ambient air pollution and depression: a systematic review with meta-analysis up to 2019. Science of The Total Environment. 2020;701:134721.
19. Diseases GoCaPMoMN-C. In: survey NCDNn, editor. Addis Ababa, Ethiopian Federal Democratic Republic of Ethiopia Ministry of
Health AND Ethiopian Public Health Association. 2016.
20. Higgins JP, Thompson SG. Quantifying heterogeneity in a meta- analysis. Statistics in medicine. 2002;21(11):1539-58.
21. J I. Interpretation of tests of heterogeneity and bias in metaanalysis. . J Eval Clin Prac. 2008;14(5):951 –7.
22. Mufunda J, Mebrahtu G, Usman A, Nyarango P, Kosia A, Ghebrat Y, et al. The prevalence of hypertension and its relationship with obesity: results from a national blood pressure survey in Eritrea. 2006;20(1):59-65.
23. Majumdar U, Nanyonga Clarke R, Moran A, Doupe P, Gadikota-Klumpers D, Gidio A. Hypertension screening, prevalence, treatment, and control at a large private hospital in Kampala, Uganda: A retrospective analysis. PLOS global public health. 2022; 2 (5): e0000386.
24. Dejenie M, Kerie S, Reba K. Undiagnosed hypertension and associated factors among bank workers in Bahir Dar City, Northwest, Ethiopia, 2020. A cross-sectional study. PloS one. 2021;16(5):e0252298.
25. Mengistu Y. The Prevalence and Associated Factors of Undiagnosed Hypertension Among Attendants of Tibebe Ghion Specialized Hospital, Bahir Dar, Ethiopia From August 15-September 15/2022 2022.

26. Essa E, Shitie D, Yirsaw MT, Wale MZ. Undiagnosed hypertension and associated factors among adults in Debre Markos town, North-West Ethiopia: A community-based cross-sectional study. SAGE Open Medicine. 2022;10:20503121221094223.
27. Getachew F, Dirar A, Solomon D. Prevalence of undiagnosed hypertension and associated factors among residents in Gulele Sub-City, Addis Ababa, Ethiopia. J Community Med Health Educ. 2018;8(590):2161-0711.1000590.
28. Wachamo D, Geleta D, Woldesemayat EM. Undiagnosed hypertension and associated factors among adults in Hawela Tula Sub-City, Hawassa, southern Ethiopia: a community-based cross-sectional study. Risk Management and Healthcare Policy. 2020;13:2169.
29. Dereje N, Earsido A, Temam L, Abebe A. Uncovering the high burden of hypertension and its predictors among adult population in Hosanna town, southern Ethiopia: a community-based cross-sectional study. BMJ open. 2020;10(10):e035823.
30. Elias S, Dadi TK. Prevalence of Undiagnosed Hypertension and Associated Factors among Adults in Mizan Aman Town, Bench Sheko Zone, Southwest Ethiopia: A Community-based Cross-sectional Study. 2022.
31. Teshome DF, Balcha SA, Ayele TA, Atnafu A, Gelaye KA. Undiagnosed hypertension and its determinants among hypertensive patients in rural districts of northwest Ethiopia: a mediation analysis. BMC Health Services Research. 2023;23(1):1-13.
32. Regea F, Birhanu A, Shibiru A. Distribution of risks and prevalence of unscreened hypertension among adults living in rural of Dano District, West Shewa, Oromia, Ethiopia, 2020: Community based cross-sectional study. Clinical Epidemiology and Global Health. 2022;13:100908.
33. Demamu H, Abinet A, Hilina G, Nega Y. Undiagnosed hypertension and its associated factors among adult people living in Southern Ethiopia: evidence from Gunchire Woreda of gurage zone. J Hypertens Manag. 2021;7(1):1-8.
34. Asemu MM, Yalew AW, Kabeta ND, Mekonnen D. Prevalence and risk factors of hypertension among adults: A community based study in Addis Ababa, Ethiopia. PloS one. 2021;16(4):e0248934.
35. Tesfaye T. Assessment of the prevalence of hypertension and associated factors among Ethiopian federal police officers Addis Ababa, Ethiopia: a community based cross-sectional study. EC Cardiology. 2017;2(6):278-86.
36. Tesfaye TD, Temesgen WA, Kasa AS, Yismaw YS. Prevalence and associated factors of hypertension in Amhara regional state city and its' surrounding rural districts: a community-based cross-sectional study. African health sciences. 2019;19(3):2580-90.
37. Bonsa F, Gudina EK, Hajito KW. Prevalence of hypertension and associated factors in Bedele Town, Southwest Ethiopia. Ethiopian journal of health sciences. 2014;24(1):21-6.
38. Gelassa FR, Birhanu A, Shibiru A, Nagari SL, Jabena DE. Undiagnosed status and associated factors of hypertension among adults living in rural of central, Ethiopia, 2020: Uncovering the hidden magnitude of hypertension. Plos one. 2022;17(12):e0277709.
39. Roba HS, Beyene AS, Mengesha MM, Ayele BH. Prevalence of hypertension and associated factors in Dire Dawa city, Eastern Ethiopia: a community-based cross-sectional study. International journal of hypertension. 2019;2019.
40. Helelo TP, Gelaw YA, Adane AA. Prevalence and associated factors of hypertension among adults in Durame Town, Southern Ethiopia. PloS one. 2014;9(11):e112790.
41. Bekele A, Gelibo T, Amenu K, Getachew T, Defar A, Teklie H, et al. The hidden magnitude of raised blood pressure and elevated blood glucose in Ethiopia: A call for initiating community based NCDs risk factors screening program. Ethiopian Journal of Health Development. 2017;31(1):362-9.

42. Asresahegn H, Tadesse F, Beyene E. Prevalence and associated factors of hypertension among adults in Ethiopia: a community based cross-sectional study. BMC research notes. 2017;10:1-8.
43. Yadecha B, Tekle F, Fetensa G, Habte A, Zeleke B. Prevalence of hypertension and its associated factors among Gimbi town residents, Ethiopia: a community-based cross-sectional study. Integrated blood pressure control. 2020:171-9.
44. Awoke A, Awoke T, Alemu S, Megabiaw B. Prevalence and associated factors of hypertension among adults in Gondar, Northwest Ethiopia: a community based cross-sectional study. BMC cardiovascular disorders. 2012;12(1):1-6.
45. Asfaw LS, Ayanto SY, Gurmamo FL. Hypertension and its associated factors in Hosanna town, Southern Ethiopia: community based cross-sectional study. BMC research notes. 2018;11:1-6.
46. Paulose T, Nkosi ZZ, Endriyas M. Prevalence of hypertension and its associated factors in Hawassa city administration, Southern Ethiopia: Community based cross-sectional study. Plos one. 2022;17(3):e0264679.
47. Esaiyas A, Teshome T, Kassa D. Prevalence of hypertension and associate risk factors among workers at Hawassa University, Ethiopia: an institution based cross sectional study. J Vasc Med Surg. 2018;6(01):2.
48. Geleta GT, Cheme MC, Roro EM. Physical, behavioral and sociodemographic determinants of hypertension among the adult population in Nekemte town, western Ethiopia: community based study. BMC Research Notes. 2019;12(1):1-6.
49. Abebe SM, Berhane Y, Worku A, Getachew A. Prevalence and associated factors of hypertension: a crossectional community based study in Northwest Ethiopia. PloS one. 2015;10(4):e0125210.
50. Shukuri A, Tewelde T, Shaweno T. Prevalence of old age hypertension and associated factors among older adults in rural Ethiopia. Integrated blood pressure control. 2019:23-31.
51. Badego B, Yoseph A, Astatkie A. Prevalence and risk factors of hypertension among civil servants in Sidama Zone, south Ethiopia. Plos one. 2020;15(6):e0234485.
52. Kebede B, Ayele G, Haftu D, Gebremichael G. The prevalence and associated factors of hypertension among adults in Southern Ethiopia. International journal of chronic diseases. 2020;2020.
53. Gudina EK, Michael Y, Assegid S. Prevalence of hypertension and its risk factors in southwest Ethiopia: a hospital-based cross-sectional survey. Integrated blood pressure control. 2013:111-7.
54. Bayray A, Meles KG, Sibhatu Y. Magnitude and risk factors for hypertension among public servants in Tigray, Ethiopia: A cross-sectional study. PloS one. 2018;13(10):e0204879.
55. Kumma WP, Lindtjørn B, Loha E. Prevalence of hypertension, and related factors among adults in Wolaita, southern Ethiopia: A community-based cross-sectional study. Plos one. 2021;16(12):e0260403.
56. Belay DG, Fekadu H, Molla MD, Chekol HA, Adugna DG, Melese E, et al. Prevalence and associated factors of hypertension among adult patients attending the outpatient department at the primary hospitals of Wolkait tegedie zone, Northwest Ethiopia. Frontiers in Neurology. 2022:1678.
57. Berhe DA, Yenit MK, Baraki AG. The burden of hypertension and associated factors among adults visiting the adult outpatient department at Yekatit 12 hospital medical College, Addis Ababa, Ethiopia, 2016. Vascular Health and Risk Management. 2020:545-52.
58. Ayalew TL, Wale BG, Zewudie BT. Burden of undiagnosed hypertension and associated factors among adult populations in Wolaita Sodo Town, Wolaita Zone, Southern Ethiopia. BMC Cardiovascular Disorders. 2022;22(1):293.

59. Bushara SO, Noor SK, Elmadhoun WM, Sulaiman AA, Ahmed MHJRf. Undiagnosed hypertension in a rural community in Sudan and association with some features of the metabolic syndrome: how serious is the situation? 2015;37(6):1022-6.
60. Omer SA, Alhamed M, Elmubarak MA, Elmubarak MA, Ibrahim RM, Ahmed FS, et al. Prevalence of undiagnosed hypertension and related risk factors in Wad-Medani Alkobra locality in Gezira State, Sudan, 2022. 2023;14(3):038-44.
61. Noor SK, Bushara SO, Sulaiman AA, Elmadhoun WMJAJoN, Transplantation. A preliminary survey of un-diagnosed hypertension among Nubians and Coptics in Atbara and Eldamer Cities, Sudan: does ethnicity affect prevalence? 2013;6(3):193-6.
62. Pengpid S, Peltzer KJTPAMJ. Undiagnosed hypertension in Sudan: results of the cross-sectional national STEPS survey in 2016. 2022;42.
63. Kilama D, Ayella DO, Asiimwe I, Nakibuuka B, Laker F, Bongomin FJIbpc. Undiagnosed Hypertension Among Market Salespersons in Kitgum Central Market, Northern Uganda. 2023:37-46.
64. Bosu WK, Reilly ST, Aheto JMK, Zucchelli E. Hypertension in older adults in Africa: a systematic review and meta-analysis. PloS one. 2019;14(4):e0214934.
65. Sarki AM, Nduka CU, Stranges S, Kandala N-B, Uthman OA. Prevalence of hypertension in low-and middle-income countries: a systematic review and meta-analysis. Medicine. 2015;94(50).
66. Pyakurel P, Yadav DK, Thapa J, Thakur N, Sharma P, Koirala N, et al. Prevalence and associated risk factor of hypertension among individuals of age 18-59 years in South-eastern Nepal: a cross-sectional study. Nepalese Heart Journal. 2019;16(1):19-26.
67. Anchala R, Kannuri NK, Pant H, Khan H, Franco OH, Di Angelantonio E, et al. Hypertension in India: a systematic review and meta-analysis of prevalence, awareness, and control of hypertension. Journal of hypertension. 2014;32(6):1170.
68. Shah N, Shah Q, Shah AJ. The burden and high prevalence of hypertension in Pakistani adolescents: a meta-analysis of the published studies. Archives of public health. 2018;76:1-10.
69. Ataklte F ES, Kaptoge S, Taye B, Echouffo-Tcheugui JB, Kengne AP:. Burden of undiagnosed
hypertension in sub-saharan Africa: a systematic review and meta-analysis. Hypertension. 2015;65 (2):291–8.
70. Ramirez LA, Sullivan JC. Sex Differences in Hypertension: Where We Have Been and Where We Are Going. Am J Hypertens. 2018;31(12):1247-54.
71. Chikhladze N. Effektivnost'terapii lerkanidipinom pri arterial'noy gipertenzii u pozhilykh. Systemic Hypertension. 2012;9(3):29-33.
72. Wamala JF, Karyabakabo Z, Ndungutse D, Guwatudde D. Prevalence factors associated with hypertension in Rukungiri district, Uganda-a community-based study. African health sciences. 2009;9(3).
73. Houinato DS, Gbary A, Houehanou Y, Djrolo F, Amoussou M, Segnon-Agueh J, et al. Prevalence of hypertension and associated risk factors in Benin. Revue d'épidémiologie et de Santé Publique. 2012;60(2):95-102.
74. Mosca L, Linfante AH, Benjamin EJ, Berra K, Hayes SN, Walsh BW, et al. National study of physician awareness and adherence to cardiovascular disease prevention guidelines. circulation. 2005;111(4):499-510.
75. Wizner B, Grodzicki T, Gryglewska B, Gasowski J, Kocemba J. Knowledge about hypertension and blood pressure level. Przeglad lekarski. 2000;57(7-8):402-5.

76. De Ramirez SS, Enquobahrie D, Nyadzi G, Mjungu D, Magombo F, Ramirez M, et al. Prevalence and correlates of hypertension: a cross-sectional study among rural populations in sub-Saharan Africa. Journal of human hypertension. 2010;24(12):786-95.
77. Soubeiga JK, Millogo T, Bicaba BW, Doulougou B, Kouanda S. Prevalence and factors associated with hypertension in Burkina Faso: a countrywide cross-sectional study. BMC Public Health. 2017;17:1-8.
78. Oda E, Kawai R. Body mass index is more strongly associated with hypertension than waist circumference in apparently healthy Japanese men and women. Acta diabetologica. 2010;47:309-13.
79. Sum G ea. Patients with More Comorbidities Have Better Detection of Chronic Conditions, but Poorer Management and Control 2020.
80. Mega TA, Dabe NE. Khat (Catha Edulis) as a risk factor for cardiovascular disorders: systematic review and meta-analysis. The Open Cardiovascular Medicine Journal. 2017;11:146.
81. Patel N. Mechanism of action of cathinone: the active ingredient of Khat (Catha Edulis. East African medical journal. 2000;77(6).
82. Brenneisen R, Fisch H, Koelbing U, Geisshusler S, Kalix P. Amphetamine- like effects in humans of the khat alkaloid cathinone. British journal of clinical pharmacology. 1990;30(6):825-8.
83. Balint EE, Falkay G, Balint GA. Khat–a controversial plant. Wiener Klinische Wochenschrift. 2009;121:604-14.
84. Bertakis KD, Azari R. The influence of obesity, alcohol abuse, and smoking on utilization of health care services. FAMILY MEDICINE-KANSAS CITY-. 2006;38(6):427.
85. Howes L, Reid J. The effects of alcohol on local, neural and humoral cardiovascular regulation. Clinical Science. 1986;71(1):9-15.
86. Birlew T, Alemseged F. Risk factors for hypertension among adults. An analysis of survey data on chronic non-communicable disease at Gilgel gibe field research center, south west Ethiopia. Science. 2015;3(2):281-90.
87. Muhihi AJ, Anaeli A, Mpembeni RN, Sunguya BF, Leyna G, Kakoko D, et al. Prevalence, awareness, treatment, and control of hypertension among young and middle-aged adults: results from a community-based survey in rural Tanzania. International journal of hypertension. 2020;2020.
88. Lu J, Lu Y, Wang X, Li X, Linderman GC, Wu C, et al. Prevalence, awareness, treatment, and control of hypertension in China: data from 1· 7 million adults in a population-based screening study (China PEACE Million Persons Project). The Lancet. 2017;390(10112):2549-58.
89. Lv X, Niu H, Qu Y, Li M, Li L, Ma X, et al. Awareness, treatment and control of hypertension among hypertensive patients aged 18 to 59 years old in the northeast of China. Scientific reports. 2018;8(1):17019.

Click here to access/download;Figure;Table 2 charactere of the articles.docx

Table 2. Characteristics of the included studies in the horn of Africa.

S.No	Author	Publication year	Country	setting	quality	Sample size	Prevalence
1	Dejenie M etal (24)	2020	Ethiopia	urban	low risk	513	24.80%
2	Mengistu Y (25)	2022	Ethiopia	urban	low risk	191	25.10%
3	Essa E etal (26)	2022	Ethiopia	urban	low risk	600	12.70%
4	Getachew F ethal (27)	2018	Ethiopia	urban	low risk	422	13.25%
5	Wachamo D etal (28)	2019	Ethiopia	urban	low risk	383	12.30%
6	Dereje N etal (29)	2020	Ethiopia	urban	low risk	634	6.90%
7	Elias S, Dadi TK (30)	2022	Ethiopia	urban	low risk	738	14.80%
8	Teshome DF etal (31)	2023	Ethiopia	rural	low risk	2436	84.00%
9	Regea F etal (32)	2022	Ethiopia	rural	low risk	605	14.60%
10	Mogas SB etal (15)	2021	Ethiopia	urban	low risk	915	21.10%
11	Demamu H etal (33)	2021	Ethiopia	rural	low risk	574	45.30%
12	Asemu MM etal (34)	2021	Ethiopia	urban	low risk	3560	19.29%
13	Tesfaye T (35)	2021	Ethiopia	urban	low risk	584	24.30%
14	Tesfaye TD (36)	2019	Ethiopia	mixed urban and rural	low risk	1405	5.33%
15	Bonsa F ethal (37)	2014	Ethiopia	urban	low risk	396	9.34%
16	Gelassa FR etal (38)	2022	Ethiopia	rural	low risk	605	21.32%
17	Roba HS etal(39)	2019	Ethiopia	urban	low risk	903	12.60%
18	Helelo TP etal (40)	2014	Ethiopia	urban	low risk	518	9.10%
19	Bekele A etal (41)	2017	Ethiopia	mixed urban and rural	low risk	9788	15.14%
20	Asresahegn H, (42)	2017	Ethiopia	urban	low risk	487	18.10%

21	Yadecha B etal (43)	2020	Ethiopia	urban	low risk	471	24.84%
22	Awoke A.etal (44)	2012	Ethiopia	urban	low risk	679	10.60%
23	Asfaw LS etal (45)	2018	Ethiopia	urban	low risk	524	5.40%
24	Paulose T etal (46)	2022	Ethiopia	urban	low risk	612	9.00%
25	Essayas A etal (47)	2018	Ethiopia	urban	low risk	618	7.3%
26	Geleta GT etal (48)	2019	Ethiopia	urban	low risk	705	16.59%
27	Abebe SM etal (49)	2015	Ethiopia	mixed urban and rural	low risk	2141	23.31%
28	Shukuri A etal (50)	2019	Ethiopia	rural	low risk	401	35.91%
29	Badego B etal (51)	2020	Ethiopia	urban	low risk	575	3.82%
30	Kebede B etal (52)	2020	Ethiopia	urban	low risk	784	31.76%
31	Gudina EK etal (53)	2013	Ethiopia	mixed urban and rural	low risk	734	8.58%
32	Bayray A etal (54)	2018	Ethiopia	urban	low risk	1523	5.31%
33	Kumma WP etal (55)	2021	Ethiopia	urban	low risk	2483	9.34%
34	Belay DG etal (56)	2022	Ethiopia	mixed urban and rural	low risk	432	28.70%
35	Berhe DA etal (57)	2020	Ethiopia	urban	low risk	414	17.39%
36	Ayalew. TL etal (58)	2022	Ethiopia	Urban	Low risk	644	28.8%
37	J Mufunda et al (22)	2006	Ertirea	Mixed urban and rural	Low risk	2352	12.7%
38	S.O. Bushara et al (59)	2015	Sudan	Rural	Low risk	1099	38.2%

39	Sawsan A. etal (60)	2023	Sudan	Mixed urban and rural	Low risk	464	10.1%
40	Sufan K etal (61)	2013	Sudan	Urban	Low risk	170	38.2%
41	Supa Pengpid et al.(62)	2022	Sudan	Mixed urban rural	Low risk	2057	26.2%
42	Kilama et al (63)	2023	Uganda	Mixed urban rural	Low risk	240	16.7%
43	Usnish Majumdar etal (23)	2022	Uganda	Urban	Low risk	17,777	19%

Click here to access/download;Figure;Table 3 predictors of undiagnosed HTN.docx

Variable		OR(95%CI)	Heterogeneity (I^2,P-value)	Total studies	Sample size
Sex	Male	1.81(1.42, 2.3)*	72.5%%, 0.001	8	3914
	Female	1	1		
Monthly income	<2000 ETB	0.71(0.51,1.0)	0%, 0.331	2	1174
	>2000ETB	1	1		
Age	18-34	1	1		
	35-44	1.88(1.5,2.37)*	22.5%, 0.266	6	5353
	>=45	2.49(2.09,2.97)*	0%, 0.702	9	7841
Regular exercise	Yes	1	1		
	No	2.26(1.54,3.32)*	64.3%, 0.023	5	2846
Excess salt consumption	Yes	1.19(0.58, 2.47)	87.4%, 0.005	2	990
	No	1	1		
Distance	<35min	0.52(0.35,0.79)*	88.6%, 0.003	2	1210
	35>=min	1	1		
Knowledge	Good	1	1		
	Poor	3.29(2.39, 4.53)*	45.7%, 0.137	4	2297
BMI	Normal	3.84(2.96,4.98)*	96.1%,<0.001	4	2430
	overweight	2.11(1.57,2.87)*	76.7%,<0.001	7	5695
	Underweight	1	1		
Family history of hypertension	Yes	1.13(0.84,1.5)	88%, <0.001	6	5374
	No	1	1		
	Yes	2.16(1.47,3.16)*	94.8%,<0.001	2	1343
	No	1	1		
Occupation	Housewife	1.84(0.97,3.48)	88.8%,0.008	2	1022
	Merchant	0.819(0.43,1.53)	0%, 0.673	2	1022
	Unemployed	1.14(0.65,1.97)	14.4%,0.32	4	2273
	Government employed	1	1		
Health seeking	Good	1	1		
	Poor	2.79(2.01, 3.86)*	0%, 0.646	4	2174
Vegetable consumption	Low	1.99(1.36,2.91)*	61%,0.036	5	2663
	Not-consume	1.23(0.88,1.83)	58.2%,0.091	3	1540
	Normal	1	1		
Smoking	Yes	1.47(1.13,1.93)*	91.5%,<0.001	5	3166
	No	1	1		
triglyceride level	High	1.58(1.32,1.89)*	35.1%, 0.202	4	4221
	Normal	1	1		
Chat chewing	Yes	2.18(1.54,3.09)*	89.7%,<0.001	3	1987
	No	1	1		

Alcohol drinking	Yes	1.75(1.32,2.33)	77.2%,0.002	5	5023
	No	1	1		

Table 3. Summary of factors associated with undiagnosed hypertension in the horn of Africa, 2023

Note: * significantly associated with undiagnosed hypertension.

Table-1: a searching strategy for the study undiagnosed hypertension in the horn of Africa.

MeSH Heading	Synonyms	Entry terms	Combination	# Of article	Last date of searching	Electronic Data bases & webs
Undiagnosed hypertension	High blood pressure	undiagnosed hypertension, hypertension, high blood pressure, Ethiopia, Eritrea, Sudan, Kenya, Uganda, Djibouti, Somali, South Sudan	Undiagnosed hypertension OR hypertension OR high blood pressure AND Ethiopia OR Djibouti OR Eritrea OR Somali OR Uganda OR Sudan OR South Sudan OR Kenya	87,779	January 23, 2023	Medline /PubMed
			Undiagnosed hypertension OR hypertension OR high blood pressure AND Ethiopia OR Djibouti OR Eritrea OR Somali OR Uganda OR Sudan	134,371	January 28, 2023	EMBASE

			OR South Sudan OR Kenya			
				324	February , 20, 2023	Google
				25,300	February 30, 2023	Google Scholar

Click here to access/download;Figure;Figure 2 pooled prevalence.docx

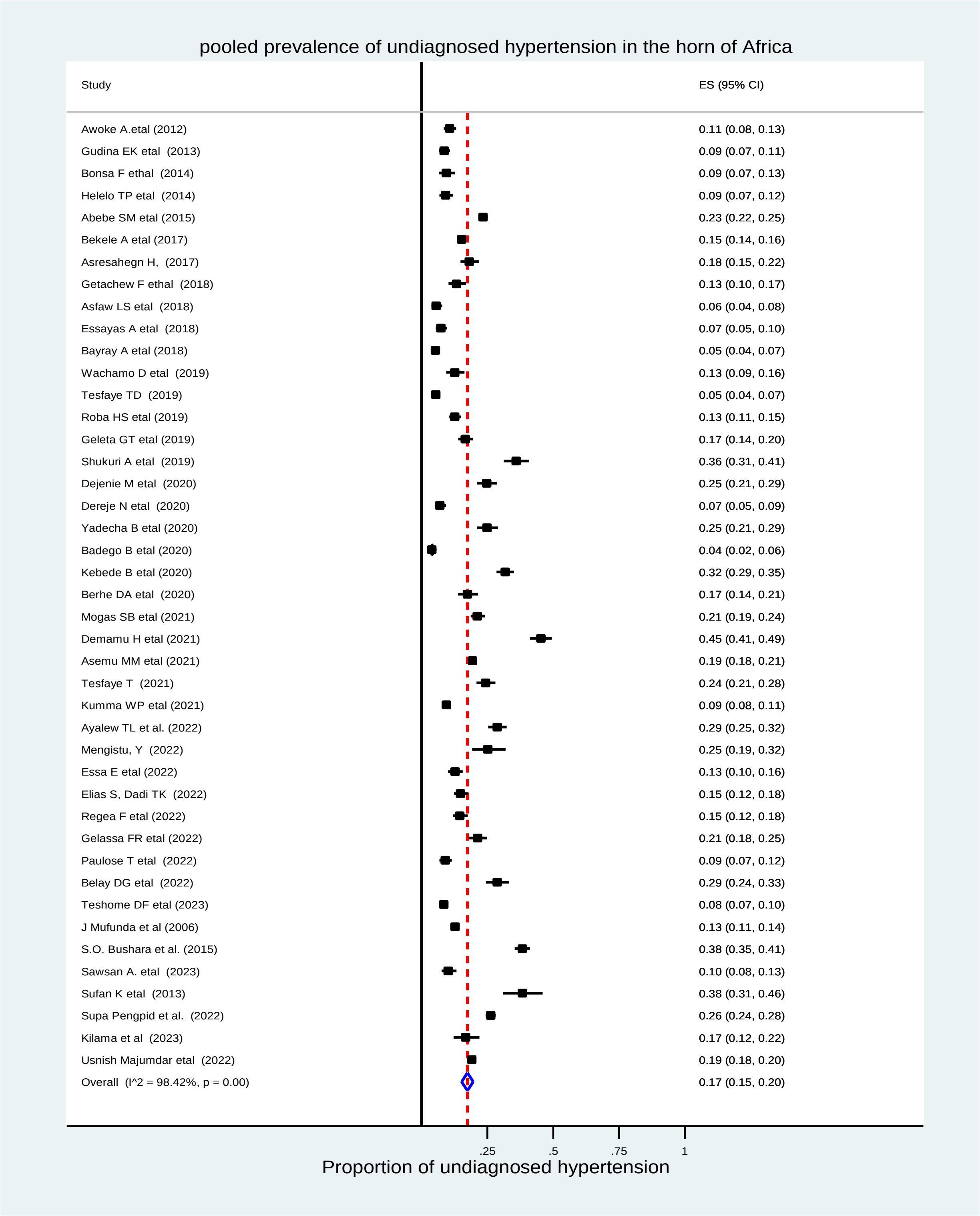

Figure-2: The pooled prevalence of undiagnosed hypertension in the horn of Africa, 2023.

Click here to access/download;Figure;Figure 4 undiagnosed hypertension by country.docx

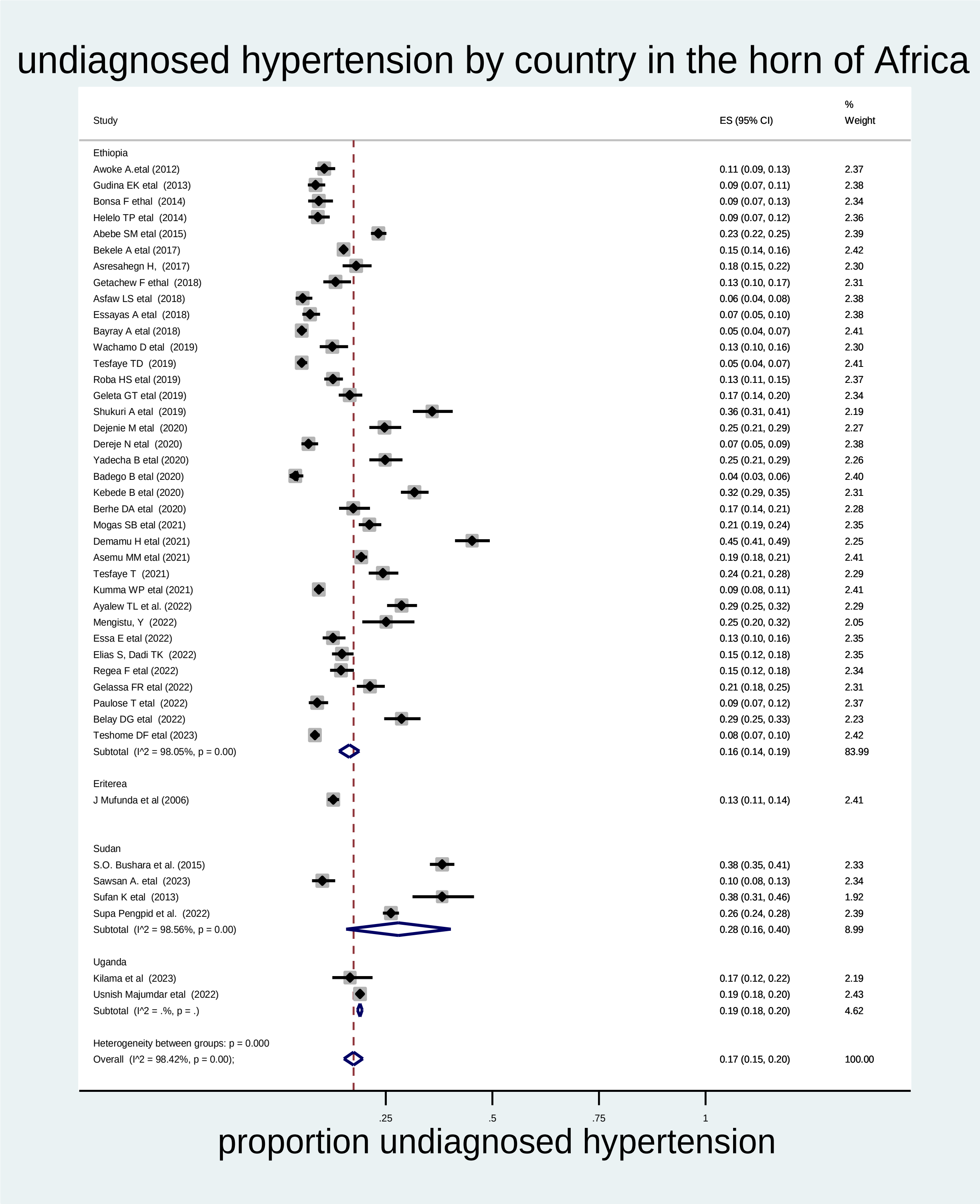

Figure-4: The pooled prevalence of undiagnosed hypertension in the horn of Africa.by country, 2023

Click here to access/download;Figure;Figure 5 undiagnosed hypertensionby setting.docx

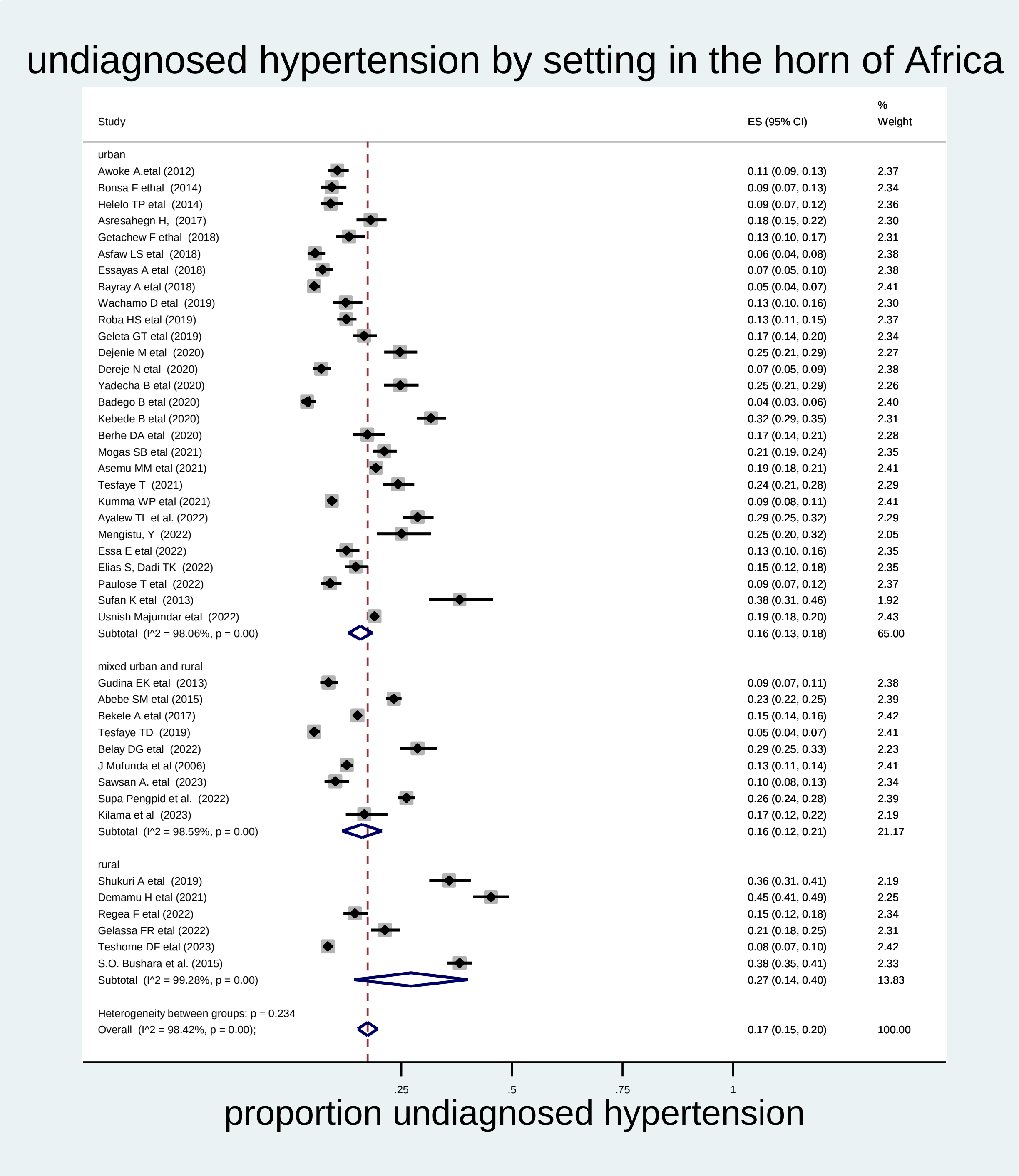

Figure-5: The pooled prevalence of undiagnosed hypertension in the horn of Africa by study setting, 2023.

Click here to access/download;Figure;Figure 6 trend of undiagnosed hypertension.docx

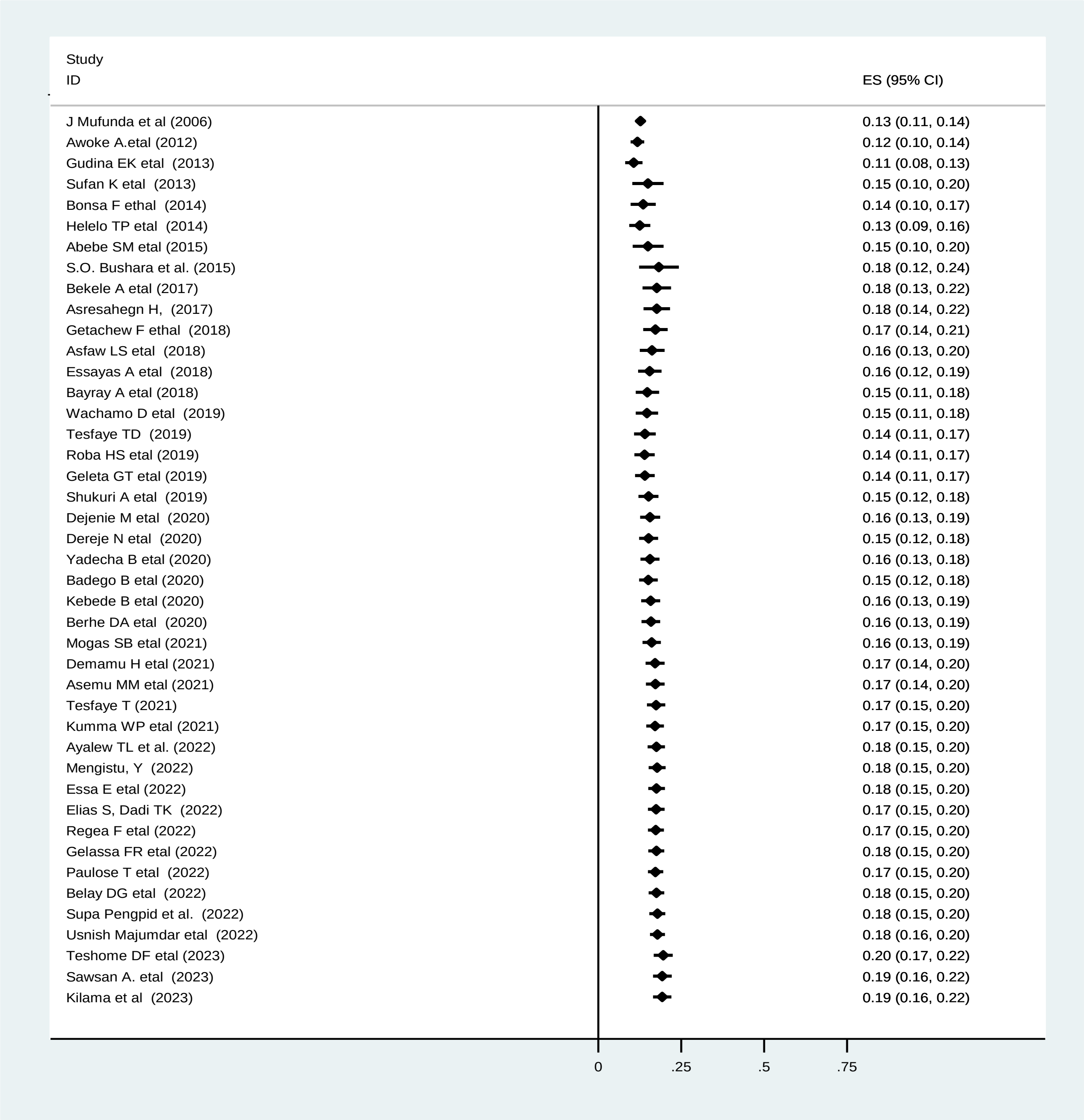

Figure-6: the trend of undiagnosed hypertension in the horn of Africa

Click here to access/download;Figure;Figure 3 funnel plot.docx

Number of studies = 43 Root MSE = 7.947

Std_Eff	Coefficient	Std. err.	t	P>|t|	[95% conf.	interval]
slope	.1202394	.0185998	6.46	0.000	.0826764	.1578024
bias	3.157316	2.194321	1.44	0.158	-1.274205	7.588836

Test of H0: no small-study effects P = 0.158

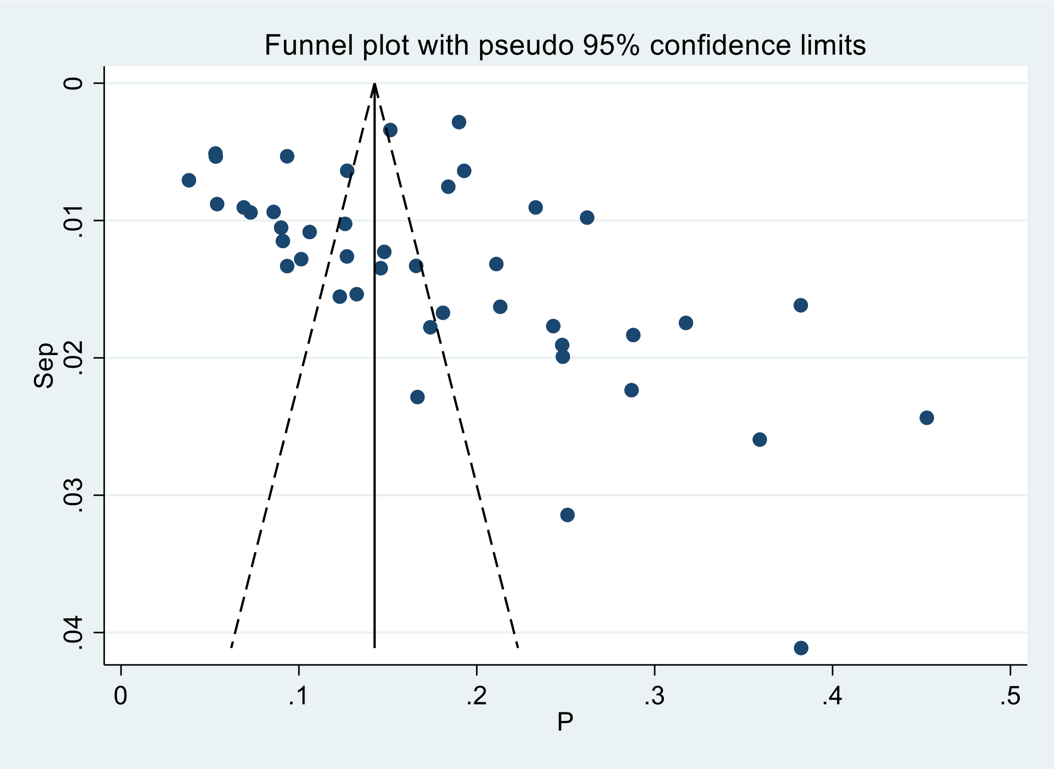

Figure-3: funnel plot of undiagnosed hypertension in the horn of Africa

Click here to access/download;Figure;Figure 1 Prisma.docx

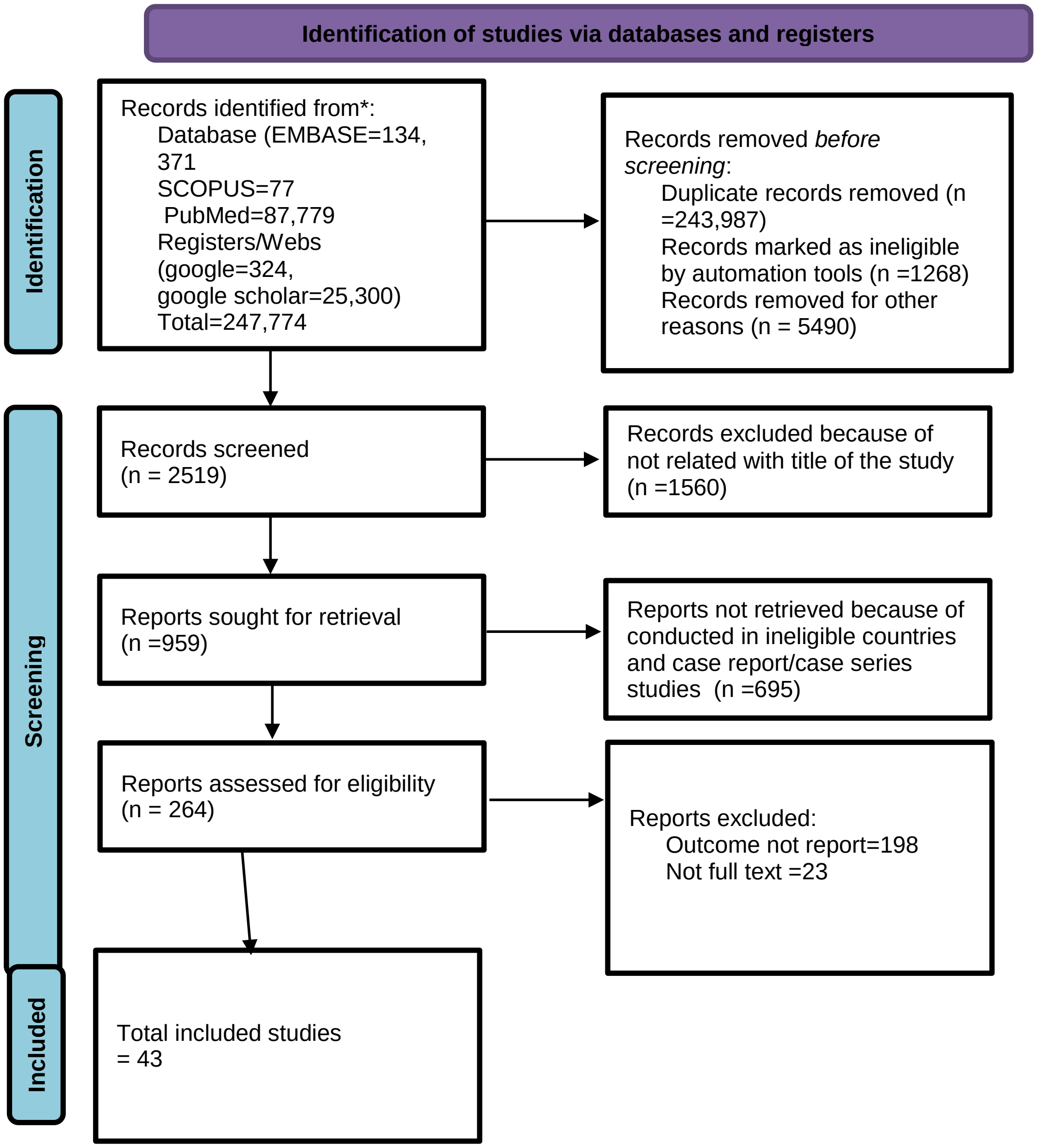

Figure-1 PRISMA flow diagram of study selection for the trend, prevalence and determinants of undiagnosed hypertension in the horn of Africa, 2023.

Click here to access/download

Supporting Information

STROBE-checklist HTN.docx

Click here to access/download
Supporting Information
PRISMA_2020_checklist.docx

Click here to access/download
Supporting Information
S-2 (supporting figures) factors.docx

Click here to access/download
Supporting Information
S-1 (supporting file-1) sensetivity.docx
