# Supplementary material for: Trend, burden and determinants of undiagnosed hypertension in the Horn of Africa: A systematic review and meta-analysis
Source: PLoS One. 2024 Aug 23;19(8):e0303940. doi: 10.1371/journal.pone.0303940 (PMC11343413; doi:10.1371/journal.pone.0303940)
Supplement: Attachment — Submitted filename: respose to reviewers.pdf [file pone.0303940.s008.pdf]

Trend, burden and determinants of undiagnosed hypertension in the horn of Africa: A systematic review and meta-analysis.

Muluken Chanie Agimas*[1], Nebiyu Mekonnen Derseh[1], Fantu Mamo[1], Moges Tadesse Abebe[2] Tilahun Yemanu[1], Meron Asmamaw[1].

[1] Department of Epidemiology and Biostatistics, institute of public health, college of medicine and health science, university of Gondar, Gondar, Ethiopia.

[2]Department of Nursing, College of Health Science, Debark University, Debark, Ethiopia

Authors address:

Muluken Chanie Agimas (MC): mulukensrc12@gmail.com

Nebiyu Mekonnen Derseh (NMD): nebiyumek12@gmail.com

Fantu mamo(FM): fantuma3@gmail.com

Moges Tadesse Abebe (MTA): moges7045@gmail.com

Tilahun Yemanu(TY): yemanu.tilahun@gmail.com

Meron Asmamaw(MA): merryalem101@gmail.com

Corresponding Author

MC: mulukensrc12@gmail.com

To: PLOS ONE <em@editorialmanager.com>

Manuscript Number: PONE-D-24-00667

Cover Letter for reviewer's comments and authors responses

We would like to thank both the Editorial board members and reviewers for kind comments and suggestions. Accordingly, we have tried to correct and respond to those comments point by point, page by page, and line by line. Therefore, we prepared this author's response, the revised manuscript, the separate revised figures and tables, and the tracked changes of the manuscript.

Sincerely, with best regards!

Muluken Chanie Agimas

ID: https://orcid.org/0000-0003-0808-124X

Comments from review and editorial board	Authors response
**Reviewers comment**	
You have listed distance from the health facility, regular exercise, age, sex, knowledge, BMI, health seeking behavior, low vegetable consumption, smoking, high triglyceride levels (TGL), chat chewing, and alcohol consumption as risk factors for the higher prevalence of undiagnosed hypertension. While it is true that factors such as distance from the health facility, age, sex, knowledge, BMI, and health seeking behavior can contribute to both the decrease or increase of undiagnosed rates and the prevalence of hypertension, I have a question regarding the	Dear reviewer Thank you so much for your detail and excellent view, and we are lucky to have you review my assignment. We took a lesson from your view and your constructive comments. You asked the most important issue, which we also challenged during data extraction. As you have suggested, such variables seem to be a factor in hypertension instead of undiagnosed

role of regular exercise, low vegetable consumption, smoking, high TGL, chat chewing, and alcohol consumption in causing the higher prevalence of undiagnosed hypertension. Although these factors are known risk factors for the development of hypertension, they may not directly cause a person to remain undiagnosed. Therefore, it might be necessary to revise the factors that specifically contribute to the occurrence of undiagnosed hypertension, given that one of your research objectives is to identify the factors influencing the prevalence of undiagnosed hypertension.	hypertension. We feel that such variables could potentially be a factor in hypertension. We have had an extensive discussion about such variables. Some of the team members suggested that as long as the primary research reports this variable as a predictor for undiagnosed hypertension, we have to show the pooled effect of such variables. Secondly, this team member also suggested that such variables have an indirect effect on undiagnosed hypertension. For example, people with no regular exercise may have a sedentary lifestyle, including not seeking treatment and diagnosis as early as possible. Additionally, people with

	alcohol consumption, chat chewers who do not control their body weight, smokers', and high-fat food consumers probably have a sedentary lifestyle. This behaviour equally affects their early treatment and diagnosis-seeking ehavior. On the contrary, people who have a healthy lifestyle may seek treatment and diagnosis early. They follow and implement the health providers' advice. People with normal body weight may be assumed to not be at risk for hypertension. Some people believe that hypertension is for fat people. This may negatively affect their behaviour in terms of early detection and treatment. Therefore, missing or

	excluding these variables can be considered a bias, and the articles may not convey the full information or not reflect the true pooled effect size of the previous primary articles. Such variables have an indirect effect on undiagnosed hypertension. But the other team members argued that the previous authors in the primary research included such variables wrongly, which we have to remove from the analysis. Finally, after a detailed discussion, we concluded and supported the idea that these variables have an indirect effect on undiagnosed hypertension and that removing these variables is a source of bias. Dear reviewer, your concern was

	also the concern of the team. Finally, we solved the problem as stated above.
It is possible that there are other determining factors, such as cultural practices or beliefs, socioeconomic factors etc. in addition to the ones you mentioned, that contribute to the undiagnosed status.	Thank you, dear editor. You are right; such a variable may have an effect on undiagnosed hypertension. But as far as our searching went, we didn't find such variables. Some of the variables were non-significant after the pooled analysis.
It might be better to include "horn of Africa" among your key words rather than **"Ethiopia."**	Dear reviewer, Sorry, it was an editorial error. And thus, a necessary amendment has been made.
You have to make it I^2 not I2.	Dear reviewer, We accepted the comment, and we revised accordingly.
How may the less than 35-minute journey time to a medical facility lead to an underdiagnosis of hypertension? Kindly give this some further	Dear reviewer Thank you so much for your excellent view. An amendment has been made.

thought. in particular, the symbol "<". Rewriting it to read as follows would make it clearer: “a trip time to a medical institution of more than 35 minutes may be a factor in the high rate of undiagnosed hypertension.”	
In your conclusion section you said, less than 35 minutes of travel time was found to be protective against undiagnosed hypertension. Do you believe that in the case of undiagnosed hypertension, it is reasonable to say protective unprotective? Being undiagnosed itself is a problem. I believe it would be more acceptable to say either raise or decrease the rate of undiagnosed.	That is great view. We accept the comment and amendment has made accordingly.

**Introduction section:**<br>1. Nine million ischemic heart disease and 3.5 million strokes can be attributed to high blood pressure. lines 77–78 on page 4. Is it every year? Please explain. Additionally, ischemic should be used instead of ischaemic.<br>2. Space out the word **And determinats** on page 5, line 96.	Dear reviewer, It was an annual case. Regarding the spelling and space between words, we have corrected accordingly.
**Methodology section:**<br>**Design:**<br>1. Describe your study design here first, and then you can mention as you used the PRISMA-P 2015 Guidelines to report it not to design.	Dear reviewer, a necessary amendment has been made accordingly.
**Searcing strategy:**<br>1. Make it searching not searcing.	Dear reviewer, an amendment has been made accordingly.

2. Using the search terms like "undetected hypertension, systolic blood pressure and/or diastolic blood pressure" may have increased the quantity of papers you found for review.	Dear reviewer Thank you so much for your suggestion. The entry term was obtained from MeSH term synonyms. We were trying to search using the entry term you mentioned. But it has lower numbers of articles than the entry term """Undiagnosed hypertension OR hypertension OR high blood pressure AND Ethiopia OR Djibouti OR Eritrea OR Somali OR Uganda OR Sudan OR South Sudan OR Kenya." This entry term provides the highest number of articles than any other entry term. The reason why we used the entry term hypertension was because some articles report the prevalence of undiagnosed hypertension in the reported hypertensive cases. Our eligible articles include not only undiagnosed hypertension. But also articles conducted on hypertension. Because, as we stated earlier, some articles report the prevalence of undiagnosed hypertension in

	the reported hypertensive cases. That is why we used the broad term to reduce the risk of missing the articles. Dear reviewer, we understand your concern. But we try too many options to avoid the risk of missing articles. For example, we search articles from a list of references, from grey literature, and separate searching in each country. That is why we found 247,774 searched-for articles. No searching entry term provides more than this number of articles.
**Data analysis:**<br><br>1. Please remove the word "anal" on page 8, line 161.	Sorry for the spelling error. Now it has been corrected.
**Result section:**	

1. What does it mean a **crossconditional** study design? On page 9, line 177.	Dear reviewer It was an editorial problem. It means that a cross-sectional study design instead of crossconditional study design.
2. Please spaced out it “Egger’sstatistical” on page 9, 190	Dear reviewer, an amendment has been made accordingly.
3. Give space for “factor(Table-3, S-2).” On page 11, line 234.	Dear reviewer, an amendment has been made accordingly.
4. Make subscript the word “was” **there** was **no**. on page 12, line 248	Dear reviewer, an amendment has been made accordingly.

Reviewer two	Authors response
Search Strategy and Databases: The inclusion of Google and Google Scholar raises reproducibility issues. Prioritizing peer-reviewed databases would enhance scientific rigor.	Dear reviewer It is great. The reason we include many searching websites, engines, and databases is to reduce the risk of missing articles. You are right: as the number of searches in databases and engines increases, the number of articles will be very large and duplicated. But duplication is better than missing an article. Because in this study, we include all articles that report the outcome of interest. If we were used only for the prioritised peer reviews, In databases, we miss many articles that are very important for our study. Different books say that searching on more than five search engines is highly

	recommended to conduct a comprehensive search. Otherwise, the reported number of articles will be questionable. Dear reviewer, that is why we used many search engines.

Study Selection and Quality Appraisal: While the approach of paired author screening is robust, a detailed account of disagreements and their resolutions would add transparency to the selection process.	Dear reviewer, you are right. Paired-author screening may have disagreements between the authors and between the paired groups. But this disagreement is very important for the quality of the searching and article selection processes. This disagreement provides a good opportunity for further discussion, re-evaluation, and reading. The discussion and further reading can improve the quality of the work.

Eligibility Criteria and Outcomes: The clear definition of inclusion/exclusion criteria and outcomes is a strength. Further elaboration on the identification and exclusion of studies not reporting relevant outcomes would be beneficial.	Dear reviewer Thank you so much for your constructive comment again. You are right. Adding what you suggested makes the article more scientifically valuable. Thus, amendments have been made accordingly.
Data Extraction: The methodology for data extraction and discrepancy resolution among authors should be described in more detail to underscore the review's systematic nature.	Dear reviewer Thank you so much for your interesting comments. We try to narrate what we have done. If it needs detailed clarification, amendments have been made.
Data Analysis: The heterogeneity assessment and the choice of modeling require a deeper discussion. Exploring the impact of study design heterogeneity on the pooled effect size is crucial.	Dear reviewer, Initially, we plan to incorporate all study designs. Such as cross-sectional, cohort, case control, and experimental study designs. But all searched articles were conducted by a cross-sectional study and included in the current systematic review and meta-

	analysis. Thus, there is no more risk of heterogeneity because of the differences in study design. Regarding the detailed narration about choosing the model, an amendment has been made accordingly in the method section lines 176–188.
Duplicate Records: The high volume of duplicates suggests a need for a more precise search strategy or a review of the deduplication process. Utilizing EndNote's advanced deduplication features could improve accuracy.	Dear reviewer That is great, and thank you so much for your impressive idea. You are right; it might be a possible reason. But the high number of duplicated articles in our work was because we used many search engines. We used Google, Google Scholar, PubMed/Medline, EMBASE, SCOPUS, and the published articles' reference lists. The articles generated from these searching engines were

	exported to one endnote library. Which increases the number of duplicated articles. Dear reviewer, we tried all possible entry terms, but the most comprehensive and maximum number of articles were generated using the following entry term: “Undiagnosed hypertension OR hypertension OR high blood pressure AND Ethiopia OR Djibouti OR Eritrea OR Somali OR Uganda OR Sudan OR South Sudan OR Kenya." If we used the entry term “horn of Africa," we definitely missed many articles. Because published articles have no such term. Articles are conducted in a specific setting. Therefore, as the number of generated articles increases,

	the number of duplicates will also increase. This is what happened in our study.
Search Period and Typographic Errors: The mention of "February 30, 2023," along with other typographical errors, undermines the manuscript's precision and professionalism. Thorough proofreading is imperative.	Dear reviewer, Thank you so much for your suggestion. Based on your recommendation, we try to correct accordingly.

Thank you, dear editor and reviewers, for your great scientific contribution!
