# Supplementary material for: Trend, burden and determinants of undiagnosed hypertension in the Horn of Africa: A systematic review and meta-analysis
Source: PLoS One. 2024 Aug 23;19(8):e0303940. doi: 10.1371/journal.pone.0303940 (PMC11343413; doi:10.1371/journal.pone.0303940)
Supplement: Attachment — Submitted filename: HPN.pdf [file pone.0303940.s009.pdf]

Trend, burden, and determinants of undiagnosed hypertension in the Horn of Africa: A systematic review and meta-analysis.

Deleted: h

Muluken Chanie Agimas*[1], Nebiyu Mekonnen Derseh[1], Fantu Mamo[1], Moges Tadesse Abebe[2] , Tilahun Yemanu[1], Meron Asmamaw[1].

[1] Department of Epidemiology and Biostatistics, institute of public health, college of medicine and health science, university of Gondar, Gondar, Ethiopia.

[2]Department of Nursing, College of Health Science, Debark University, Debark, Ethiopia

Authors address:

Muluken Chanie Agimas (MC): mulukensrc12@gmail.com

Nebiyu Mekonnen Derseh (NMD): nebiyumek12@gmail.com

Fantu mamo(FM): fantuma3@gmail.com

Moges Tadesse Abebe (MTA): moges7045@gmail.com

Tilahun Yemanu(TY): yemanu.tilahun@gmail.com

Meron Asmamaw(MA): merryalem101@gmail.com

Corresponding Author

MC: mulukensrc12@gmail.com

Abstract

Background: Hypertension is a silent killer disease that rarely shows signs and symptoms. Because of this nature, most people do not seek medical care early. The global report revealed that half of the world's population lives with undiagnosed hypertension. Among the people who are living with hypertension, half of them are unaware of their status. In the Horn of Africa, hypertension is becoming a major public health issue. However there is no systematic review and meta-analysis to assess the trend, prevalence, and determinants of undiagnosed hypertension and provide conclusive and tangible evidence about the problem.

Objective: To assess the trend, burden, and determinates of undiagnosed hypertension in the Horn of Africa.

Method: Published and unpublished papers were searched on Google, Google Scholar, PubMed/Medline, EMBASE, SCOPUS, and the published article's reference list. The searching mechanism was established using Medical Subject Heading (MeSH) terms by combining the key terms of the title. To assure the quality of the included articles, Joana Brigg's Institute critical appraisal checklist was used, and those articles with a total quality score greater than 50% were considered to have a low risk of bias. A sensitivity test was conducted to evaluate the heterogeneity of the studies. The visual funnel plot test and Egger's and Begg's statistics in the random effect model were done to evaluate the publication bias and small study effect. The I^2 statistics were also used to quantify the amount of heterogeneity between the included studies.

Commented [BA1]: Shorten the abstract to about 300 words. Merge the objective to background in the abstract section

Commented [BA2]: In this background, please write about undiagnosed hypertension in the horn of Africa. you can use this for the background and objective" The global report revealed that half of the world's population lives with undiagnosed hypertension. The problem is expected to be worse in low-income countries such as in Horn of Africa countries. We planned to determine the trend, burden, and determinates of undiagnosed hypertension in this region and provide conclusive and tangible evidence for interventions".

Deleted: But

Result: A total of 43 studies were included for analysis, and the pooled prevalence of undiagnosed hypertension in the Horn of Africa was 17% (95% CI: 15–20). A trip time to a medical institution of less than 35 minutes (OR=0.52, 95%CI: 0.35-0.79), no regular exercise (OR=2.26, 95% CI: 1.54-3.32), age >=45 years (OR =2.51, 95% CI: 1.66-3.8), age 35-44 years (OR =1.88, 95% CI: 1.5-2.37), male (OR =1.72, 95% CI: 1.34–2.2), poor knowledge (OR=3.29, 95%CI: 2.39,4.53), normal BMI (OR=3.84, 95% CI: 2.96-4.98), Overweight (OR=1.97, 95% CI: 2.96-4.98), poor health seeking (OR= 2.79, 95%CI: 2.01-3.86), low vegetable consumers (OR= 1.99, 95%CI:1.36-2.91), smoking (OR= 1.47, 95%CI: 1.13-1.93), high triglyceride (OR=1.83, 95%CI:1.33-2.52), chat chewing (OR=2.18, 95%CI: 1.54-3.09), and alcohol drinking (OR=1.75, 95%CI: 1.32-2.33) were the determinants of undiagnosed hypertension.

Conclusion and recommendation: The pooled prevalence of undiagnosed hypertension was low in the Horn of Africa. A trip time to a medical institution of less than 35 minutes may be a factor in the low rate of undiagnosed hypertension. Whereas no regular exercise, age >=45 years, age 35-44 years, male, poor knowledge, normal BMI, overweight, poor health, low vegetable consumption, smokers, high triglyceride, chat chewing, and alcohol drinking were the risk factors of undiagnosed hypertension. Therefore, health professionals and higher officials should promote a healthy lifestyle, encourage early health-seeking behavior, and expand screening programs.

Keywords: undiagnosed hypertension, Horn of Africa, meta-analysis, systematic review

Commented [BA3]: Where is the treated. Three outcomes you should write here , burden , trend and factors

Deleted: determinats

Commented [BA4]: Do not repeat the result part here. you can summarize these one. You can say “individual level variables were identified that affect the undiagnosed hypertension”. Trend also important to mention here.

Deleted: behaviour

Deleted: me

Introduction

Hypertension can be defined by two or more repeated measurements of systolic blood pressure ≥ 130mm Hg or ≥ 80 for diastolic pressure [1]. Hypertension is a silent killer disease that rarely shows signs and symptoms. Because of the nature of the disease, most people do not seek medical care and go to health institutions when the disease becomes more advanced or complicated [2]. Unmanaged hypertension leads to stroke, premature death, heart disease, chronic kidney disease, and other causes of mortality [3]. Approximately one billion people have developed hypertension around the globe, and if essential intervention is not given, by 2025, hypertension will have increased to 1.5 billion cases [4]. The global report revealed that half of the world's population lives with undiagnosed hypertension [5]. Among the people who are living with hypertension, half of them are unaware of whether they are living with hypertension or not [6]. Overall, increased blood pressure can cause 9 million ischemic heart diseases and 3.5 million strokes each year [7].

Of the global hypertension cases, Africa contributes about 46% [8]. Furthermore, the specific death rate of hypertension in Africa accounts for 25% of the total deaths [9]. Even though the burden of hypertension is high, most African countries cannot afford the health service costs because there are other prioritized health conditions and an inadequate supply of drugs and other medical equipment [10]. Nations around the world, including the Horn of Africa, have been working to meet the target of Sustainable Development Goal Target 3.4 of reducing the premature mortality rate of non-communicable disease by one-third through early prevention and control in 2030 [11].

Deleted: can not

Deleted: prioritised

Undiagnosed hypertension in the Horn of Africa is becoming a major public health agenda. Evidence shows that hypertension has become a hidden epidemic [12, 13]. Undiagnosed hypertension can be attributed to or associated with smoking, an unhealthy diet (for example, high intake of salt, fat, and refined sugar stress), a lack of regular physical activity, a body mass index greater than 25, older age, and alcohol drinking or an unhealthy lifestyle [14, 15]. African countries have been working to reduce the health and economic impact of hypertension, but still, the magnitude of the problem is rising. Furthermore, there is no updated information about the burden, trend, and prevalence of undiagnosed hypertension in the Horn of Africa. Thus, this systematic review and meta-analysis aimed to try to determine the trend, prevalence, and determinate factors of undiagnosed hypertension in the Horn of Africa.

Deleted: e aim of this systematic review and meta-analysis was

Methodology

Design

Though we planned to include all types of study design, all searched articles were conducted using cross-sectional studies and included for analysis for this study. The Preferred Reporting Items for Systematic Reviews and Meta-Analysis Protocols (PRISMA-P 2015 Guidelines) were used [16].

Searching strategy

All the important published and unpublished papers were searched on Google, Google Scholar, PubMed/Medline, EMBASE, and SCOPUS databases, and the published article's reference list from January 23, 2023, to February 30, 2023. The searching mechanism was established using Medical Subject Heading (MeSH) terms by combining the key terms of

the title. Again, reviewing the published article reference list was used to identify those papers that were missing on Google, Google Scholar, PubMed/Medline, and EMBASE. Content experts were advised to obtain more papers or to reduce the risk of missing them. Six authors (Muluken Chanie Agimas, Meron Asmamaw, Moges Tadesse Abebe, Nebyu Mekonnen Derseh, Fantu Mamo, and Tilahun Yemanu) searched the articles independently, cross-checking their results. This systematic review and meta-analysis were conducted by extracting studies in the context of the prevalence of undiagnosed hypertension using the following key entry terms. "Undiagnosed hypertension OR hypertension OR high blood pressure AND Ethiopia OR Djibouti OR Eritrea OR Somali OR Uganda OR Sudan OR South Sudan OR Kenya" **(Table-1)**.

Commented [BA5]: Please write their name in Abbreviations

Deleted: was

Deleted: hypertension OR

Commented [BA6]: Table 1 should be submitted as supplementary.

Table-1: A search strategy for the study of undiagnosed hypertension in the Horn of Africa.

MeSH Heading	Synonyms	Entry terms	Combination	# Of article	Last date of searching	Electronic Data bases & webs
Undiagnosed hypertension	High blood pressure	undiagnosed hypertension, hypertension, high blood pressure, Ethiopia, Eritrea, Sudan, Kenya, Uganda, Djibouti, Somali, South Sudan	Undiagnosed hypertension OR hypertension OR high blood pressure AND Ethiopia OR Djibouti OR Eritrea OR Somali OR Uganda OR Sudan OR South Sudan OR Kenya	87,779	January 23, 2023	Medline/PubMed

Deleted: a

Deleted: searching

Deleted: hypertention

Deleted: horn

Deleted: Undiagnosed hypertension

Deleted: blood pressure

			Undiagnosed hypertension OR hypertension OR high blood pressure AND Ethiopia OR Djibouti OR Eritrea OR Somali OR Uganda OR Sudan OR South Sudan OR Kenya	134,371	January 28, 2023	EMBASE
				324	February , 20, 2023	Google
				25,300	February 30, 2023	Google Scholar

Deleted: ¶

Deleted: ¶

Study selection and quality appraisal

The retrieved articles were imported into EndNote X8 software. The duplicated articles were removed after all articles were imported into one database with endnote X8. The title and abstracts of each article were screened by paired authors (MC and NMD). The discrepancy among the paired authors was solved by discussion and other review teams (MA, FM, DA, TY, and MTA). Articles with full text were considered for systematic and meta-analysis. To assure the quality of the retrieved and included articles, Joana Briggs's Institute critical appraisal checklist for simple prevalence [17] and analytical cross-sectional studies [18]

Nine and eight checklist items, respectively, were considered. Articles with a total quality score of more than 50% were labeled as paper-qualified articles, indicating a low risk of bias [18]. During the quality appraisal, these discrepancies were solved by discussion.

Eligible criteria

Inclusion criteria

The authors screened all the papers' titles, abstracts, and full texts. Then:

- Those articles report the outcome of interest, namely the prevalence of undiagnosed hypertension or factors associated with undiagnosed hypertension.
- All cross-sectional, prospective cohort studies, case-control studies, and inter-sectional studies were considered.
- Those articles are published in English and published at any time.
- Both published and unpublished articles were incorporated.
- Studies conducted or published at any time were included in the study.

Exclusion Criteria

The authors critically, comprehensively, independently, and blindly screen the abstracts and the full texts. After this extensive screening, the authors excluded the following articles:

- Articles that did not report the outcome of interest, namely the prevalence of undiagnosed hypertension or factors associated with undiagnosed hypertension, were excluded.
- Case reports and case series studies were excluded.

Commented [BA7]: Please change this phrase, this vague. This not clear whether you planned to included only low risk. Make it clear

Deleted: l

Commented [BA8]: The quality assessment score of each study should be submitted as supplementary in table.

Deleted: Eligibly

Commented [BA9]: Are studies community based or included institutional a also?

Commented [BA10]: Why prospective court only? why nor retrospective

Deleted: case control

Commented [BA11]: What does this study design mean?

- Articles that didn't have full text were excluded because of difficulties in quality assessment.

Outcomes of the study

The primary outcome of the intended systematic and meta-analysis was the prevalence of undiagnosed hypertension and associated factors in the Horn of Africa.

Commented [BA12]: Is the trend not your outcome ?

Operational definition

Undiagnosed hypertension: was defined as systolic blood pressure of 140 mmHg and above and/or diastolic blood pressure of 90 mmHg and above, without previous history or anti-hypertensive treatment during the survey diagnosis [19].

Commented [BA13]: How do you manage if the different studies used different cut point for HPN diagnosis? Is all used the same cut point

Deleted: hypertention

Data extraction

Next to the exhaustive data searching and screening, eligible articles were extracted using a data extraction format that includes the name of the first author, publication year, study area or region, study design, tool, quality of the articles, number of samples, number of undiagnosed hypertension, proportions, standard error of the proportion, odds ratio for each independent variable, confidence interval of the odds ratio, and standard error of each odds ratio. These were extracted from each study using a Microsoft Excel spreadsheet. To extract the data, the JBI tool was adopted. The three paired authors extracted the data independently using the same standard format and then cross-checked the consistency of the extracted data. The cross-checking of the extracted data includes the consistency of all the data extracted from the three groups of paired authors. For example, the consistency of the quality score of each article, the number of samples for each article, the proportions of undiagnosed hypertension in each article, the standard error of the proportion, the odds ratio for each independent variable, the confidence interval of the odds ratio, and the standard error of each odds ratio. The discrepancy, such as the inconsistency in the quality assessment of the paper, was solved by discussion between the three paired teams. The majority agreement was not solved by group consensus.

Deleted: For the purpose of extracting

Data analysis

Data from the eligible articles was entered into Microsoft Excel and then exported to Stata 14 for further data analysis. The I^2 statistics, or the index of heterogeneity, was used to quantify the amount of heterogeneity between the included studies [20]. Values of I^2 of 25%, 50%, and 75% were considered low, medium, and high heterogeneity, respectively

[21]. The significance of the heterogeneity between the studies was evaluated by a p-value of I^2 less than 0.05. Sensitivity analysis and subgroup analysis using the study setting and regions were conducted to evaluate and manage the heterogeneity. The visual funnel plot test (visual method) and Egger's statistics (objective/statistical technique) in the random effect model were done to evaluate the publication bias or small study effect. Additionally, to manage the heterogeneity between the studies, a random effect model was used. The random effect model assumes that the true treatment effects in the individual studies may be different from each other. It is not only variation within the study but also variation across the study. To consider this variation, we used the random effect model. But the fixed effect model should be used when there is little evidence of heterogeneity. The effect size was reported by OR with its 95% confidence level to estimate the statistical association between undiagnosed hypertension and associated factors. The prevalence of undiagnosed hypertension and other factors was reported in both the text and the forest plot.

Results

Out of 247,774 searched articles, 243,987 were excluded because of duplicated articles. About 2519 records were screened, and 264 of them were assessed for eligibility. Finally, 43 potential studies have been included for systematic review and meta-analysis, as summarized in the PRISMA flow diagram **(Figure 1).**

Figure-1 PRISMA flow diagram of study selection for the trend, prevalence, and determinants of undiagnosed hypertension in the Horn of Africa, 2023.

Characteristics of included studies

All included studies were conducted using a cross-sectional study design. Of the total included studies, the majority, 28 (65.1%) were conducted in an urban setting. The total

Deleted: assumption of the random effect model i

Deleted: ,

Deleted: summarised

Deleted: -

Deleted: horn

Commented [BA14]: The majority of the study were from Ethiopia, be sure that there was no researching bias.

number of participants included or used for analysis was 64,156, and the minimum and maximum sample sizes used in the studies were 170 [22] and 17,777 [23] respectively. **(Table 2).**

Table 2. Characteristics of the included studies in the Horn of Africa.

S.No	Author	Publication year	Country	setting	quality	Sample size	Prevale[illegible]
1	Dejenie M *et al* [24]	2020	Ethiopia	urban	low risk	513	24.80%
2	Mengistu Y [25]	2022	Ethiopia	urban	low risk	191	25.10%
3	Essa E etal [26]	2022	Ethiopia	urban	low risk	600	12.70%
4	Getachew F ethal [27]	2018	Ethiopia	urban	low risk	422	13.25%
5	Wachamo D etal [28]	2019	Ethiopia	urban	low risk	383	12.30%
6	Dereje N etal [29]	2020	Ethiopia	urban	low risk	634	6.90%
7	Elias S, Dadi TK [30]	2022	Ethiopia	urban	low risk	738	14.80%
8	Teshome DF etal [31]	2023	Ethiopia	rural	low risk	2436	84.00%
9	Regea F etal [32]	2022	Ethiopia	rural	low risk	605	14.60%
10	Mogas SB etal [15]	2021	Ethiopia	urban	low risk	915	21.10%
11	Demamu H etal [33]	2021	Ethiopia	rural	low risk	574	45.30%
12	Asemu MM etal [34]	2021	Ethiopia	urban	low risk	3560	19.29%
13	Tesfaye T [35]	2021	Ethiopia	urban	low risk	584	24.30%
14	Tesfaye TD [36]	2019	Ethiopia	mixed urban and rural	low risk	1405	5.33%
15	Bonsa F ethal [37]	2014	Ethiopia	urban	low risk	396	9.34%
16	Gelassa FR etal [38]	2022	Ethiopia	rural	low risk	605	21.32%
17	Roba HS etal[39]	2019	Ethiopia	urban	low risk	903	12.60%

Commented [BA15]: The tittle is not complete, Pease add, undiagnosed HPN
Included, the study setting in the table, community or institutional

Deleted: horn

Commented [BA16]: Prevalence of what?

Commented [BA17]: Please write in italic format the *etal*

Formatted: Font: Italic

Formatted: Font: Italic

Commented [BA18]: Correct it

18	Helelo TP etal [40]	2014	Ethiopia	urban	low risk	518	9.10%
19	Bekele A etal [41]	2017	Ethiopia	mixed urban and rural	low risk	9788	15.14%
20	Asresahegn H, [42]	2017	Ethiopia	urban	low risk	487	18.10%
21	Yadecha B etal [43]	2020	Ethiopia	urban	low risk	471	24.84%
22	Awoke A.etal [44]	2012	Ethiopia	urban	low risk	679	10.60%
23	Asfaw LS etal [45]	2018	Ethiopia	urban	low risk	524	5.40%
24	Paulose T et al [46]	2022	Ethiopia	urban	low risk	612	9.00%
25	Essayas A etal [47]	2018	Ethiopia	urban	low risk	618	7.3%
26	Geleta GT etal [48]	2019	Ethiopia	urban	low risk	705	16.59%
27	Abebe SM etal [49]	2015	Ethiopia	mixed urban and rural	low risk	2141	23.31%
28	Shukuri A etal [50]	2019	Ethiopia	rural	low risk	401	35.91%
29	Badego B etal [51]	2020	Ethiopia	urban	low risk	575	3.82%
30	Kebede B etal [52]	2020	Ethiopia	urban	low risk	784	31.76%
31	Gudina EK etal [53]	2013	Ethiopia	mixed urban and rural	low risk	734	8.58%
32	Bayray A etal [54]	2018	Ethiopia	urban	low risk	1523	5.31%
33	Kumma WP etal [55]	2021	Ethiopia	urban	low risk	2483	9.34%
34	Belay DG etal [56]	2022	Ethiopia	mixed urban and rural	low risk	432	28.70%

35	Berhe DA etal [57]	2020	Ethiopia	urban	low risk	414	17.39%
36	Ayalew. TL etal [58]	2022	Ethiopia	Urban	Low risk	644	28.8%
37	J Mufunda et al [22]	2006	Eritrea	Mixed urban and rural	Low risk	2352	12.7%
38	S.O. Bushara et al [59]	2015	Sudan	Rural	Low risk	1099	38.2%
39	Sawsan A. etal [60]	2023	Sudan	Mixed urban and rural	Low risk	464	10.1%
40	Sufan K etal [61]	2013	Sudan	Urban	Low risk	170	38.2%
41	Supa Pengpid et al.[62]	2022	Sudan	Mixed urban rural	Low risk	2057	26.2%
42	Kilama et al [63]	2023	Uganda	Mixed urban rural	Low risk	240	16.7%
43	Usnish Majumdar etal [23]	2022	Uganda	Urban	Low risk	17,777	19%

The pooled prevalence of undiagnosed hypertension in the Horn of Africa.

In the random effects model, the pooled prevalence of undiagnosed hypertension in the Horn of Africa was 17% (95% CI, 15%–20%), and the heterogeneity among the studies was statistically significant ($I^2 = 98.42\%$, P-value < 0.001) **(Figure-2).**

Figure 2: The pooled prevalence of undiagnosed hypertension in the Horn of Africa, 2023.

Assessment of publication bias

Egger's statistical test evidenced that there was no publication bias among the included studies (β = 7.947, P-value = 0.158) **(Figure-3).**

Deleted: Ertirea

Deleted: horn

Deleted: Figure-2

Deleted: horn

Commented [BA19]:

Commented [BA20R19]: Remove the table above the figure 2

Figure 3: Funnel plot of undiagnosed hypertension in the Horn of Africa

Handling heterogeneity

In the random effect model of pooled prevalence, statistically significant heterogeneity was detected. To solve this problem, sensitivity analysis and subgroup analysis were performed.

Sensitivity analysis

To manage the influence of a single study in meta-analysis estimation (to assess the heterogeneity), the random effects model and sensitivity analysis were conducted, and no study excessively influenced the overall pooled prevalence of undiagnosed hypertension (**S-1**).

Subgroup analysis by country

In the subgroup analysis by country, the highest prevalence of undiagnosed hypertension was observed in Sudan (28%; 95% CI: 16%–40%), and the lowest prevalence of undiagnosed hypertension was reported in Eritrea (13% 95% CI: 11%–14%) **(Figure-4).**

Figure 4: The pooled prevalence of undiagnosed hypertension in the Horn of Africa.by country, 2023

Subgroup analysis by study setting

Subgroup analysis was also carried out in the study setting as a source of heterogeneity. The highest prevalence of undiagnosed hypertension was observed in rural areas (27%; 95% CI: 14%–40%) (**Figure 5**).

Figure-5: The pooled prevalence of undiagnosed hypertension in the Horn of Africa by study setting, 2023.

Trends of undiagnosed hypertension in the Horn of Africa

The trend of undiagnosed hypertension in the Horn of Africa increased over time, from 13% in 2006 to 20% in 2023 (**Figure 6**).

Deleted: Figure-3
Deleted: f
Deleted: h
Deleted: heterogenity
Deleted: there was no study that excessively influenced
Commented [BA21]: Only one study was included from Eretria. You included it in the subgroup analysis. For subgroup at least two study should be included in each group. Please remove the Eretria from subgroup
Deleted: Ertirea
Deleted: Figure-4
Deleted: h
Deleted: hypertension
Deleted: by study setting was observed in
Deleted: Figure-5
Deleted: h
Deleted: horn
Commented [BA22]: Is this change significant , please indicate int
Deleted: Figure-6

Figure 6: The trend of undiagnosed hypertension in the Horn of Africa

Factors associated with undiagnosed hypertension

As briefly described in Table 3, sex, age, BMI, chat chewing, alcohol consumption, regular exercise, knowledge about hypertension, health-seeking behavior, vegetable consumption, level of triglyceride, and time to reach the health facilities were statistically significant factors for undiagnosed hypertension in the Horn of Africa. From the random effect model estimate, the pooled odds of undiagnosed hypertension among males were 1.81 times higher than those of females (OR =1.81, 95% CI: 1.42–2.3), with I^2 =72.5% and a P-value of 0.001. Egger's test indicates that there was no small study effect (P-value = 0.454), and the random effects model reported no single study that excessively influences the effect size of this factor (**Table 3**, **S-2**).

Another significant factor for undiagnosed hypertension was age, and thus age >= 45 years and 35-44 years was 2.49 (OR = 2.49, 95% CI: 2.09-2.97), (I^2 =0%, P-value=0.72), and 1.88 (OR = 1.88, 95% CI: 1.5-2.37, I^2 =22.5%, P-value=0.266) times higher risk for undiagnosed hypertension than 18-34 years, respectively. The Egger's test indicates that there was no small study effect in age >=45 years (P-value =0.444) and 35-44 years (P = 0.502). This means that in the random effects model, there was no single study that excessively influenced the effect size of this factor (**Table 3, S-2).**

The pooled effect size of undiagnosed hypertension among those who had no regular exercise was 2.2 times more likely than the counterpart (OR = 2.26, 95% CI: 1.54–3.32, I^2 =64.3%, P-value =0.023). Egger's test indicates that there was no small study effect (P-

Deleted: Figure-6

Deleted: t

Deleted: horn

Deleted: behaviour

Commented [BA23]: What dose this mean? Is it supplementary? where is S-1.
S-2 included many figures group as S-2. Please label each figure as s-1, s-2...... and special in each main text accordingly. Apply for the rest below

Deleted: Table-3

Deleted: Table-3

value = 0.524), and in the random effect model, there was no single study that excessively influenced the effect size of this factor (Table 3, **S-2).**

Deleted: Table-3

The pooled odds of undiagnosed hypertension in a trip time to a medical institution of less than 35 minutes were reduced by 48% compared to those above 35 minutes to reach the health facilities (OR = 0.52, 95% CI: 0.35-0.79, I^2= 88.6%, P-value =0.003). Egger's test indicates that there was no small study effect (P-value = 0.245), and in the random effects model, there was no single study that excessively influenced the effect size of this factor (Table 3, **S-2).**

Deleted: Table-3

Deleted: ¶

Regarding knowledge of hypertension, adults who had poor knowledge about hypertension were 3.29 times more likely to have undiagnosed hypertension than those with good knowledge about hypertension (OR = 3.29, CI: 2.39, 4.53), with I^2 = 43.7% and a P-value of 0.137. Egger's test indicates that there was no small study effect (P-value = 0.95), and thus there was no single study that excessively influenced the effect size of this factor **(Table-3, S-2).**

Again, the odds of undiagnosed hypertension among normal BMI and overweight were 3.84 (OR=384, 95% CI: 2.96-4.98, I^2=96.1%, P-value=<0.001) and 2.11 (OR=2.11, CI: 1.57-2.83, I^2=76.7%, P-value=<0.001) times higher than underweight. Egger's test indicates that there was no small study effect for normal BMI (P-value =0.549), for overweight (P-value=0.139), and thus there was no single study that excessively influences the effect size of this factor **(Table-3, S-2).**

Having poor health-seeking behaviour was 2.79 times more likely to have undiagnosed hypertension than having good health-seeking behaviour (OR = 2.79, CI: 2.01-3.86, I^2 =

0%, P-value =0.646). Egger's test also indicates that there was no small study effect (P-value = 0.236), and thus there was no single study that excessively influenced the pooled estimate of this factor **(Table-3, S-2).**

Another factor that influences undiagnosed hypertension is vegetable consumption. Thus, the odds of undiagnosed hypertension among low-vegetable consumers were 1.99 times higher than those of normal consumers (OR = 1.99, 95% CI: 1.36-2.91, I^2 = 61%, P-value = 0.036). Egger's test indicates that there was no small study effect (P-value = 0.928), and thus there was no single study that excessively influenced the effect size of this factor **(Table-3, S-2).**

Additionally, the odds of undiagnosed hypertension among smokers were 1.47 times higher than nonsmokers (OR = 1.47, 95% CI: 1.13–1.93, I^2 = 91.5%, P-value<0.001). Egger's test indicates that there was no small study effect (P-value = 0.715), and thus there was no single study that excessively influenced the effect size of this factor **(Table-3, S-2).** Having a high level of triglyceride was 1.58 times more likely to put you at risk for undiagnosed hypertension than a normal level of triglyceride (OR = 1.58, 95% CI: 1.31–1.89, I^2 = 35.1%, P-value = 0.202). Egger's test showed that there was no small study effect (P-value = 0.795), and thus there was no single study that excessively influenced the effect size of this factor **(Table-3, S-2).**

The odds of undiagnosed hypertension among chat chewers were 2.18 times higher than their counterparts (OR = 2.18, CI: 1.54–3.09, I^2 = 89.7%, P-value<0.001). Egger's test indicates that there was no small study effect (P-value = 0.836), and thus there was no single study that excessively influenced the effect size of this factor **(Table-3, S-2).**

On the other hand, the odds of undiagnosed hypertension among alcohol drinkers were 1.75 times higher than their counterparts (OR = 1.75, 95% CI: 1.32-2.33, I^2 = 77.2%, P-value = 0.002). Egger's test also showed that there was no small study effect (P-value = 0.743), and thus there was no single study that excessively influenced the effect size of this factor (**Table 3, S-2).**

Deleted: Table-3

Table 3. Summary of factors associated with undiagnosed hypertension in the Horn of Africa., 2023

Deleted: h

Variable		OR(95%CI)	Heterogeneity (I^2,P-value)	Total studies	Sample size
Sex	Male	1.81(1.42, 2.3)*	72.5%%, 0.001	8	3914
	Female	1	1		
Monthly income	<2000 ETB	0.71(0.51,1.0)	0%, 0.331	2	1174
	>2000ETB	1	1		
Age	18-34	1	1		
	35-44	1.88(1.5,2.37)*	22.5%, 0.266	6	5353
	>=45	2.49(2.09,2.97)*	0%, 0.702	9	7841
Regular exercise	Yes	1	1		
	No	2.26(1.54,3.32)*	64.3%, 0.023	5	2846
Excess salt consumption	Yes	1.19(0.58, 2.47)	87.4%, 0.005	2	990
	No	1	1		
Distance	<35min	0.52(0.35,0.79)*	88.6%, 0.003	2	1210
	35>=min	1	1		
Knowledge	Good	1	1		
	Poor	3.29(2.39, 4.53)*	45.7%, 0.137	4	2297
BMI	Normal	3.84(2.96,4.98)*	96.1%,<0.001	4	2430
	Overweight	2.11(1.57,2.87)*	76.7%,<0.001	7	5695
	Underweight	1	1		
Family history of hypertension	Yes	1.13(0.84,1.5)	88%, <0.001	6	5374
	No	1	1		
	Yes	2.16(1.47,3.16)*	94.8%,<0.001	2	1343
	No	1	1		
Occupation	Housewife	1.84(0.97,3.48)	88.8%,0.008	2	1022
	Merchant	0.819(0.43,1.53)	0%, 0.673	2	1022
	Unemployed	1.14(0.65,1.97)	14.4%,0.32	4	2273
	Government employed	1	1		
Health seeking	Good	1	1		
	Poor	2.79(2.01, 3.86)*	0%, 0.646	4	2174
Vegetable consumption	Low	1.99(1.36,2.91)*	61%,0.036	5	2663
	Not-consume	1.23(0.88,1.83)	58.2%,0.091	3	1540
	Normal	1	1		
Smoking	Yes	1.47(1.13,1.93)*	91.5%,<0.001	5	3166
	No	1	1		
triglyceride level	High	1.58(1.32,1.89)*	35.1%, 0.202	4	4221
	Normal	1	1		
Chat chewing	Yes	2.18(1.54,3.09)*	89.7%,<0.001	3	1987

	No	1	1		
Alcohol drinking	Yes	1.75(1.32,2.33)	77.2%,0.002	5	5023
	No	1	1		

Discussion

In this study, an attempt has been made to assess the trend, burden, and determinate factors of undiagnosed hypertension in the Horn of Africa. Thus, the pooled prevalence of undiagnosed hypertension in the Horn of Africa was 17% (95% CI: 15–20), distance takes <35 min, no regular exercise >=45 years, 35–44 years, male, good knowledge, normal BMI, overweight, poor health seeking, low vegetable consumers, smokers, high triglyceride, chat chewing, and alcohol drinking were the factors associated with undiagnosed hypertension. The pooled prevalence of undiagnosed hypertension in the current study was lower than in a systematic and meta-analysis study conducted in Sub-Saharan Africa (30%), Nigeria. (28.9%), India (29.8%), Pakistan (26.34%), and a study in Nepal (25.1%) [14, 64-68]. This may be because of the difference in the time of study and the difference in the study setting. The other possible reason may be that most of the studies conducted and included in the current study were among the urban population. This segment of the population has better health-seeking and check-up behaviour than the rural population. In the subgroup analysis by study setting and regions, the pooled prevalence of undiagnosed hypertension was higher in rural areas, which was 25% (95% CI: 12-38). The possible reason for this difference may be that the rural population has a lower educational status, a

lack of access to health facilities, and a lack of access to the media. In return, they may have low health-seeking or health check-up behaviour [69].

This systematic meta-analysis also identifies the associated factors of undiagnosed hypertension in the Horn of Africa, and thus males were more likely to have undiagnosed hypertension than females. This finding was supported by a study done in the United States of America [70]. The possible reason for this finding may be that most of the time, women have the chance to receive various maternal health services. For example, family planning delivery, antenatal care services, and immunization services provide a good opportunity for understanding their health status [24].

The odds of undiagnosed hypertension in ages 35–44 and >=45 years were higher than in ages 18–34 years. This finding is supported by studies done in Nepal [71], Uganda [72], and Benin [73] This is due to arterial stiffness increasing as age increases. In return, it increases blood pressure, and parallelly, undiagnosed hypertension is more common in older people [71].

Physical exercise was also a significant factor for undiagnosed hypertension, and, thus, adults with no regular exercise were at higher risk of undiagnosed hypertension than regular physical exercise. This is because physical activity and/or exercise have been shown to delay the development of hypertension [74]. The other possible reason might be associated with those individuals who live a sedentary life, such as those who have no exercise and are also not seeking medical care.

This systematic review and meta-analysis also revealed that the distance from health facilities to home affects undiagnosed hypertension, and thus traveling time <35 minutes

Deleted: and

Deleted: ,

Deleted: of receiving

Deleted: immunisation

Deleted: l

reduces the risk of undiagnosed hypertension. This may be because those who are at nearby health facilities access the health service more easily than those who are far away.

Poor knowledge about hypertension is another risk factor for undiagnosed hypertension. The finding was supported by the studies conducted in Rwanda and Cracow [5, 75]. This might be because adults who have low knowledge about hypertension have a low level of screening for hypertension.

Another associated factor for undiagnosed hypertension in the Horn of Africa was BMI. Normal BMI and overweight increase the risk of undiagnosed hypertension more than underweight individuals. This is supported by studies conducted in sub-Saharan African countries [76] and a national study in Burkina Faso [77]. This might be because of a 1 kg/m^2 increase in BMI; the odds of hypertension also increased as compared to underweight (risk of undiagnosed hypertension = obese/overweight > normal > underweight) [78]. Alternatively, as the BMI increases by 1 kg/m^2, there is an increase in the risk of undiagnosed hypertension. In this case, a normal BMI is associated with a associated with a higher risk of undiagnosed hypertension as compared to an underweight (low BMI) by holding other factors constant. Again, those individuals who have a normal BMI may have a poor belief that they have a low risk of developing hypertension, which makes them less likely to visit a health institution to measure their blood pressure. Alternatively, their subjective perception of the lower risk of acquiring hypertension may prevent them from measuring their blood pressure. Even during the selective/risk group screening, these individuals may be missed.

Deleted: of

In this study, adults with low health-seeking behaviour were more likely to have undiagnosed hypertension than those with good health-seeking behaviour. This might be because adults with low health-seeking behaviour have less chance to visit health institutions, and, as a result, they do not know their hypertension status [79].

This study also showed that chat chewers are more likely to have undiagnosed hypertension. This is supported by a systematic and meta-analytic study in Ethiopia [80]. This is because chat (Khat) contains cathinone, cathine, and amphetamine, which elevate topamine in the brain [81] and this leads to elevated blood pressure [82, 83]. Another possible response might be that adults living an unhealthy life give less attention to early health seeking and screening [84].

In the current study, the pooled effect of alcohol drinking was the risk factor for undiagnosed hypertension. This is due to alcohol consumption negatively affecting the central nervous system, which increases cardiac output and affects the peripheral vascular effect, which leads to elevated blood pressure [85]. Another possible reason might be that adults living an unhealthy lifestyle are given less attention to early health-seeking and screening [84].

Another finding in the current study was that adults with low consumption of vegetables were more likely to be at risk for undiagnosed hypertension. This is because vegetables and fruit are very essential food components of a healthy diet that can prevent a variety of cardiovascular diseases, including hypertension, and WHO recommends these food items to enhance the health of the community [86].

Additionally, the current study reported that alcohol drinkers had a higher risk of undiagnosed hypertension than non-alcohol drinkers. The finding is consistent with studies

Deleted: ehavior

Deleted: due tothe fact that

Commented [BA24]: ??

Deleted: b

Deleted: the

Deleted: preasure

Deleted: the

Deleted: recommended

conducted in Tanzania [87] and China [88, 89]. This might be because adults with an unhealthy lifestyle, including alcohol drinking, have less health-care-seeking behavior [84]. Furthermore, the odds of undiagnosed hypertension were higher among adults with a high triglyceride level than a normal level of triglyceride. This might be because high triglycerides are often a sign of other conditions that increase the risk of heart disease, hypertension, and other chronic diseases, and such adults are comorbid. As a result, they seek medical care, have frequent contact with health professionals, and know their blood pressure status. The strength of the study was the involvement of many experts (data extraction by six researchers) during the searching, screening, appraisal, and data extraction processes. Research helped to make the evidence more comprehensive and valid. Furthermore, the data report adheres to the guidelines outlined in the PRISMA P-2015 statement protocol. However, some studies that categorize the variables differently and are difficult to manage were omitted.

Commented [BA25]: What are the limitations ? specially related to the hetrogenicity ?, representaiveness in the horn of Africa ?..................., definition of Cutpoint for BP categorization,

Deleted: But

Deleted: categorise

Deleted: in a different wa

Conclusion: The pooled prevalence of undiagnosed hypertension was low in the Horn of Africa. Distance takes <35 minutes was the protective factor for undiagnosed hypertension, whereas no regular exercise >=45 years, 35-44 years, being male, good knowledge, normal BMI, overweight, poor health seeking, low vegetable consumption, smokers, high triglyceride, chat chewing, and alcohol drinking were the risk factors for undiagnosed hypertension. Therefore, health professionals and higher officials should promote a healthy lifestyle, encourage early health-seeking behaviour, and expand screening programmes.

Deleted: u

Abbreviation and acronym

BMI….. Body Mass Index

CI…….Confidence Interval

OR……Odds Ratio

PRISMA…….Prefered Reporting Item for Systematic Review and Meta Analysis

WHO…….World Health Organization

Ethical declaration

Ethical approval and consent to participate

Not applicable

Consent for publication

Not applicable.

Availability of data and material.

The dataset supporting the conclusion of this article is available from the authors on request.

Competing Interest

The authors declare that they have no any financial and non-financial competing interests

Source of funding

The authors received no specific funding for this work.

Acknowledgments

The authors acknowledged University of Gondar staffs for their support.

Supporting information

S-1: sensitivity analysis of undiagnosed hypertension in the horn of Africa, 2023

S-2: Effect of each factor on undiagnosed hypertension in the horn of Africa, 2023

Author Contributions

MCA, MA, NMD and FM were responsible for conceptualization.

MCA and FM were responsible for investigation.

MCA and NMD were responsible for writing and evaluating the methodology of the research

TY, MCA, and MA were responsible for software and data analysis

MCA and MA were responsible supervision.

FM and MCA were responsible for validation

MCA, NMD and MTA were responsible for visualization.

TY, MCA, MA, FM, NMD and MTA were responsible writing – review, aproving & editing of the manscript.

Reference

1. Carey, R.M., P.K. Whelton, and A.A.H.G.W. Committee*, *Prevention, detection, evaluation, and management of high blood pressure in adults: synopsis of the 2017 American College of Cardiology/American Heart Association Hypertension Guideline.* Annals of internal medicine, 2018. **168**(5): p. 351-358.
2. Day, W., *A global brief on hypertension.* Geneva: World Health Organization, 2013.
3. Lawes, C.M., S. Vander Hoorn, and A. Rodgers, *Global burden of blood-pressure-related disease, 2001.* The Lancet, 2008. **371**(9623): p. 1513-1518.
4. Chockalingam, A., *Impact of world hypertension day.* Canadian journal of cardiology, 2007. **23**(7): p. 517-519.
5. Mazimpaka, C., et al., *Assessing the Magnitude and Risk Factors Associated With Undiagnosed Hypertension in Rural Rwanda.* Assessing the Magnitude and Risk Factors Associated With Undiagnosed Hypertension in Rural Rwanda.
6. Gulec, S., *Early diagnosis saves lives: focus on patients with hypertension.* Kidney international supplements, 2013. **3**(4): p. 332-334.
7. Williams, B., et al., *ESC/ESH Guidelines for the management of arterial hypertension [2018 ESC/ESH Guidelines for the management of arterial hypertension].* Kardiol Pol, 2019. **77**(2): p. 71-159.
8. Organization, W.H., *Global Health Observatory (GHO) data: NCD mortality and morbidity.* Retrieved August, 2016. **23**: p. 2016.
9. Organization, W.H., *Global health risks: mortality and burden of disease attributable to selected major risks*. 2009: World Health Organization.
10. Palafox, B., et al., *Wealth and cardiovascular health: a cross-sectional study of wealth-related inequalities in the awareness, treatment and control of hypertension in high-, middle-and low-income countries.* International journal for equity in health, 2016. **15**(1): p. 1-14.

11. Norheim, O.F., et al., *Avoiding 40% of the premature deaths in each country, 2010–30: review of national mortality trends to help quantify the UN Sustainable Development Goal for health.* The Lancet, 2015. **385**(9964): p. 239-252.
12. Undavalli, V.K. and H. Mp, *Prevalence of undiagnosed hypertension: a public health challenge.* Int J Community Med Public Health, 2018. **5**(4): p. 1366-1370.
13. Gerensea, H. and H. Teklay, *Trend of hypertension morbidity and mortality in Tigray Region from 2011 to 2015, Tigray, Ethiopia.* BMC research notes, 2018. **11**(1): p. 1-5.
14. Ataklte, F., et al., *Burden of undiagnosed hypertension in sub-saharan Africa: a systematic review and meta-analysis.* Hypertension, 2015. **65**(2): p. 291-298.
15. Mogas, S.B., et al., *Burden of undiagnosed hypertension among adults in urban communities of Southwest Ethiopia.* Integrated Blood Pressure Control, 2021. **14**: p. 69.
16. Moher, D., et al., *Preferred reporting items for systematic review and meta-analysis protocols (PRISMA-P) 2015 statement.* Systematic reviews, 2015. **4**(1): p. 1-9.
17. Munn, Z., et al., *Methodological guidance for systematic reviews of observational epidemiological studies reporting prevalence and cumulative incidence data.* International journal of evidence-based healthcare, 2015. **13**(3): p. 147-153.
18. Fan, S.-J., et al., *Ambient air pollution and depression: a systematic review with meta-analysis up to 2019.* Science of The Total Environment, 2020. **701**: p. 134721.
19. Diseases, G.o.C.a.P.M.o.M.N.-C., *In: survey NCDNn, editor. Addis Ababa, Ethiopian Federal Democratic Republic of Ethiopia Ministry of*
Health AND Ethiopian Public Health Association. 2016.
20. Higgins, J.P. and S.G. Thompson, *Quantifying heterogeneity in a meta-analysis.* Statistics in medicine, 2002. **21**(11): p. 1539-1558.
21. J, I., *Interpretation of tests of heterogeneity and bias in metaanalysis. .* J Eval Clin Prac, 2008. **14(5)**: p. 951 –7.
22. Mufunda, J., et al., *The prevalence of hypertension and its relationship with obesity: results from a national blood pressure survey in Eritrea.* 2006. **20**(1): p. 59-65.
23. Majumdar, U., et al., *Hypertension screening, prevalence, treatment, and control at a large private hospital in Kampala, Uganda: A retrospective analysis. PLOS global public health. 2022; 2 (5): e0000386*.
24. Dejenie, M., S. Kerie, and K. Reba, *Undiagnosed hypertension and associated factors among bank workers in Bahir Dar City, Northwest, Ethiopia, 2020. A cross-sectional study.* PloS one, 2021. **16**(5): p. e0252298.
25. Mengistu, Y., *The Prevalence and Associated Factors of Undiagnosed Hypertension Among Attendants of Tibebe Ghion Specialized Hospital, Bahir Dar, Ethiopia From August 15-September 15/2022*. 2022.
26. Essa, E., et al., *Undiagnosed hypertension and associated factors among adults in Debre Markos town, North-West Ethiopia: A community-based cross-sectional study.* SAGE Open Medicine, 2022. **10**: p. 20503121221094223.
27. Getachew, F., A. Dirar, and D. Solomon, *Prevalence of undiagnosed hypertension and associated factors among residents in Gulele Sub-City, Addis Ababa, Ethiopia.* J Community Med Health Educ, 2018. **8**(590): p. 2161-0711.1000590.
28. Wachamo, D., D. Geleta, and E.M. Woldesemayat, *Undiagnosed hypertension and associated factors among adults in Hawela Tula Sub-City, Hawassa, southern Ethiopia: a community-based cross-sectional study.* Risk Management and Healthcare Policy, 2020. **13**: p. 2169.

29. Dereje, N., et al., *Uncovering the high burden of hypertension and its predictors among adult population in Hosanna town, southern Ethiopia: a community-based cross-sectional study.* BMJ open, 2020. **10**(10): p. e035823.
30. Elias, S. and T.K. Dadi, *Prevalence of Undiagnosed Hypertension and Associated Factors among Adults in Mizan Aman Town, Bench Sheko Zone, Southwest Ethiopia: A Community-based Cross-sectional Study.* 2022.
31. Teshome, D.F., et al., *Undiagnosed hypertension and its determinants among hypertensive patients in rural districts of northwest Ethiopia: a mediation analysis.* BMC Health Services Research, 2023. **23**(1): p. 1-13.
32. Regea, F., A. Birhanu, and A. Shibiru, *Distribution of risks and prevalence of unscreened hypertension among adults living in rural of Dano District, West Shewa, Oromia, Ethiopia, 2020: Community based cross-sectional study.* Clinical Epidemiology and Global Health, 2022. **13**: p. 100908.
33. Demamu, H., et al., *Undiagnosed hypertension and its associated factors among adult people living in Southern Ethiopia: evidence from Gunchire Woreda of gurage zone.* J Hypertens Manag, 2021. **7**(1): p. 1-8.
34. Asemu, M.M., et al., *Prevalence and risk factors of hypertension among adults: A community based study in Addis Ababa, Ethiopia.* PloS one, 2021. **16**(4): p. e0248934.
35. Tesfaye, T., *Assessment of the prevalence of hypertension and associated factors among Ethiopian federal police officers Addis Ababa, Ethiopia: a community based cross-sectional study.* EC Cardiology, 2017. **2**(6): p. 278-286.
36. Tesfaye, T.D., et al., *Prevalence and associated factors of hypertension in Amhara regional state city and its' surrounding rural districts: a community-based cross-sectional study.* African health sciences, 2019. **19**(3): p. 2580-2590.
37. Bonsa, F., E.K. Gudina, and K.W. Hajito, *Prevalence of hypertension and associated factors in Bedele Town, Southwest Ethiopia.* Ethiopian journal of health sciences, 2014. **24**(1): p. 21-26.
38. Gelassa, F.R., et al., *Undiagnosed status and associated factors of hypertension among adults living in rural of central, Ethiopia, 2020: Uncovering the hidden magnitude of hypertension.* Plos one, 2022. **17**(12): p. e0277709.
39. Roba, H.S., et al., *Prevalence of hypertension and associated factors in Dire Dawa city, Eastern Ethiopia: a community-based cross-sectional study.* International journal of hypertension, 2019. **2019**.
40. Helelo, T.P., Y.A. Gelaw, and A.A. Adane, *Prevalence and associated factors of hypertension among adults in Durame Town, Southern Ethiopia.* PloS one, 2014. **9**(11): p. e112790.
41. Bekele, A., et al., *The hidden magnitude of raised blood pressure and elevated blood glucose in Ethiopia: A call for initiating community based NCDs risk factors screening program.* Ethiopian Journal of Health Development, 2017. **31**(1): p. 362-369.
42. Asresahegn, H., F. Tadesse, and E. Beyene, *Prevalence and associated factors of hypertension among adults in Ethiopia: a community based cross-sectional study.* BMC research notes, 2017. **10**: p. 1-8.
43. Yadecha, B., et al., *Prevalence of hypertension and its associated factors among Gimbi town residents, Ethiopia: a community-based cross-sectional study.* Integrated blood pressure control, 2020: p. 171-179.

44. Awoke, A., et al., *Prevalence and associated factors of hypertension among adults in Gondar, Northwest Ethiopia: a community based cross-sectional study.* BMC cardiovascular disorders, 2012. **12**(1): p. 1-6.
45. Asfaw, L.S., S.Y. Ayanto, and F.L. Gurmamo, *Hypertension and its associated factors in Hosanna town, Southern Ethiopia: community based cross-sectional study.* BMC research notes, 2018. **11**: p. 1-6.
46. Paulose, T., Z.Z. Nkosi, and M. Endriyas, *Prevalence of hypertension and its associated factors in Hawassa city administration, Southern Ethiopia: Community based cross-sectional study.* Plos one, 2022. **17**(3): p. e0264679.
47. Esaiyas, A., T. Teshome, and D. Kassa, *Prevalence of hypertension and associate risk factors among workers at Hawassa University, Ethiopia: an institution based cross sectional study.* J Vasc Med Surg, 2018. **6**(01): p. 2.
48. Geleta, G.T., M.C. Cheme, and E.M. Roro, *Physical, behavioral and sociodemographic determinants of hypertension among the adult population in Nekemte town, western Ethiopia: community based study.* BMC Research Notes, 2019. **12**(1): p. 1-6.
49. Abebe, S.M., et al., *Prevalence and associated factors of hypertension: a crossectional community based study in Northwest Ethiopia.* PloS one, 2015. **10**(4): p. e0125210.
50. Shukuri, A., T. Tewelde, and T. Shaweno, *Prevalence of old age hypertension and associated factors among older adults in rural Ethiopia.* Integrated blood pressure control, 2019: p. 23-31.
51. Badego, B., A. Yoseph, and A. Astatkie, *Prevalence and risk factors of hypertension among civil servants in Sidama Zone, south Ethiopia.* Plos one, 2020. **15**(6): p. e0234485.
52. Kebede, B., et al., *The prevalence and associated factors of hypertension among adults in Southern Ethiopia.* International journal of chronic diseases, 2020. **2020**.
53. Gudina, E.K., Y. Michael, and S. Assegid, *Prevalence of hypertension and its risk factors in southwest Ethiopia: a hospital-based cross-sectional survey.* Integrated blood pressure control, 2013: p. 111-117.
54. Bayray, A., K.G. Meles, and Y. Sibhatu, *Magnitude and risk factors for hypertension among public servants in Tigray, Ethiopia: A cross-sectional study.* PloS one, 2018. **13**(10): p. e0204879.
55. Kumma, W.P., B. Lindtjørn, and E. Loha, *Prevalence of hypertension, and related factors among adults in Wolaita, southern Ethiopia: A community-based cross-sectional study.* Plos one, 2021. **16**(12): p. e0260403.
56. Belay, D.G., et al., *Prevalence and associated factors of hypertension among adult patients attending the outpatient department at the primary hospitals of Wolkait tegedie zone, Northwest Ethiopia.* Frontiers in Neurology, 2022: p. 1678.
57. Berhe, D.A., M.K. Yenit, and A.G. Baraki, *The burden of hypertension and associated factors among adults visiting the adult outpatient department at Yekatit 12 hospital medical College, Addis Ababa, Ethiopia, 2016.* Vascular Health and Risk Management, 2020: p. 545-552.
58. Ayalew, T.L., B.G. Wale, and B.T. Zewudie, *Burden of undiagnosed hypertension and associated factors among adult populations in Wolaita Sodo Town, Wolaita Zone, Southern Ethiopia.* BMC Cardiovascular Disorders, 2022. **22**(1): p. 293.
59. Bushara, S.O., et al., *Undiagnosed hypertension in a rural community in Sudan and association with some features of the metabolic syndrome: how serious is the situation?* 2015. **37**(6): p. 1022-1026.

60. Omer, S.A., et al., *Prevalence of undiagnosed hypertension and related risk factors in Wad-Medani Alkobra locality in Gezira State, Sudan, 2022.* 2023. **14**(3): p. 038-044.
61. Noor, S.K., et al., *A preliminary survey of un-diagnosed hypertension among Nubians and Coptics in Atbara and Eldamer Cities, Sudan: does ethnicity affect prevalence?* 2013. **6**(3): p. 193-196.
62. Pengpid, S. and K.J.T.P.A.M.J. Peltzer, *Undiagnosed hypertension in Sudan: results of the cross-sectional national STEPS survey in 2016.* 2022. **42**.
63. Kilama, D., et al., *Undiagnosed Hypertension Among Market Salespersons in Kitgum Central Market, Northern Uganda.* 2023: p. 37-46.
64. Bosu, W.K., et al., *Hypertension in older adults in Africa: a systematic review and meta-analysis.* PloS one, 2019. **14**(4): p. e0214934.
65. Sarki, A.M., et al., *Prevalence of hypertension in low-and middle-income countries: a systematic review and meta-analysis.* Medicine, 2015. **94**(50).
66. Pyakurel, P., et al., *Prevalence and associated risk factor of hypertension among individuals of age 18-59 years in South-eastern Nepal: a cross-sectional study.* Nepalese Heart Journal, 2019. **16**(1): p. 19-26.
67. Anchala, R., et al., *Hypertension in India: a systematic review and meta-analysis of prevalence, awareness, and control of hypertension.* Journal of hypertension, 2014. **32**(6): p. 1170.
68. Shah, N., Q. Shah, and A.J. Shah, *The burden and high prevalence of hypertension in Pakistani adolescents: a meta-analysis of the published studies.* Archives of public health, 2018. **76**: p. 1-10.
69. Ataklte F, E.S., Kaptoge S, Taye B, Echouffo-Tcheugui JB, Kengne AP:, *Burden of undiagnosed hypertension in sub-saharan Africa: a systematic review and meta-analysis.* Hypertension, 2015. **65 (2)**: p. 291–298.
70. Ramirez, L.A. and J.C. Sullivan, *Sex Differences in Hypertension: Where We Have Been and Where We Are Going.* Am J Hypertens, 2018. **31**(12): p. 1247-1254.
71. Chikhladze, N., *Effektivnost'terapii lerkanidipinom pri arterial'noy gipertenzii u pozhilykh.* Systemic Hypertension, 2012. **9**(3): p. 29-33.
72. Wamala, J.F., et al., *Prevalence factors associated with hypertension in Rukungiri district, Uganda-a community-based study.* African health sciences, 2009. **9**(3).
73. Houinato, D.S., et al., *Prevalence of hypertension and associated risk factors in Benin.* Revue d'épidémiologie et de Santé Publique, 2012. **60**(2): p. 95-102.
74. Mosca, L., et al., *National study of physician awareness and adherence to cardiovascular disease prevention guidelines.* circulation, 2005. **111**(4): p. 499-510.
75. Wizner, B., et al., *Knowledge about hypertension and blood pressure level.* Przeglad lekarski, 2000. **57**(7-8): p. 402-405.
76. De Ramirez, S.S., et al., *Prevalence and correlates of hypertension: a cross-sectional study among rural populations in sub-Saharan Africa.* Journal of human hypertension, 2010. **24**(12): p. 786-795.
77. Soubeiga, J.K., et al., *Prevalence and factors associated with hypertension in Burkina Faso: a countrywide cross-sectional study.* BMC Public Health, 2017. **17**: p. 1-8.
78. Oda, E. and R. Kawai, *Body mass index is more strongly associated with hypertension than waist circumference in apparently healthy Japanese men and women.* Acta diabetologica, 2010. **47**: p. 309-313.
79. Sum G, e.a., *Patients with More Comorbidities Have Better Detection of Chronic*

Conditions, but Poorer Management and Control 2020.
80. Mega, T.A. and N.E. Dabe, *Khat (Catha Edulis) as a risk factor for cardiovascular disorders: systematic review and meta-analysis.* The Open Cardiovascular Medicine Journal, 2017. **11**: p. 146.
81. Patel, N., *Mechanism of action of cathinone: the active ingredient of Khat (Catha Edulis.* East African medical journal, 2000. **77**(6).
82. Brenneisen, R., et al., *Amphetamine-like effects in humans of the khat alkaloid cathinone.* British journal of clinical pharmacology, 1990. **30**(6): p. 825-828.
83. Balint, E.E., G. Falkay, and G.A. Balint, *Khat–a controversial plant.* Wiener Klinische Wochenschrift, 2009. **121**: p. 604-614.
84. Bertakis, K.D. and R. Azari, *The influence of obesity, alcohol abuse, and smoking on utilization of health care services.* FAMILY MEDICINE-KANSAS CITY-, 2006. **38**(6): p. 427.
85. Howes, L. and J. Reid, *The effects of alcohol on local, neural and humoral cardiovascular regulation.* Clinical Science, 1986. **71**(1): p. 9-15.
86. Birlew, T. and F. Alemseged, *Risk factors for hypertension among adults. An analysis of survey data on chronic non-communicable disease at Gilgel gibe field research center, south west Ethiopia.* Science, 2015. **3**(2): p. 281-290.
87. Muhihi, A.J., et al., *Prevalence, awareness, treatment, and control of hypertension among young and middle-aged adults: results from a community-based survey in rural Tanzania.* International journal of hypertension, 2020. **2020**.
88. Lu, J., et al., *Prevalence, awareness, treatment, and control of hypertension in China: data from 1· 7 million adults in a population-based screening study (China PEACE Million Persons Project).* The Lancet, 2017. **390**(10112): p. 2549-2558.
89. Lv, X., et al., *Awareness, treatment and control of hypertension among hypertensive patients aged 18 to 59 years old in the northeast of China.* Scientific reports, 2018. **8**(1): p. 17019.
