# Supplementary material for: Trend, burden and determinants of undiagnosed hypertension in the Horn of Africa: A systematic review and meta-analysis
Source: PLoS One. 2024 Aug 23;19(8):e0303940. doi: 10.1371/journal.pone.0303940 (PMC11343413; doi:10.1371/journal.pone.0303940)
Supplement: Attachment — Submitted filename: respose to reviewers for second round comments.pdf [file pone.0303940.s010.pdf]

Trend, burden and determinants of undiagnosed hypertension in the Horn of Africa: A systematic review and meta-analysis.

Muluken Chanie Agimas*[1], Nebiyu Mekonnen Derseh[1], Fantu Mamo[1], Moges Tadesse Abebe[2] Tilahun Yemanu[1], Meron Asmamaw[1].

[1] Department of Epidemiology and Biostatistics, institute of public health, college of medicine and health science, university of Gondar, Gondar, Ethiopia.

[2]Department of Nursing, College of Health Science, Debark University, Debark, Ethiopia

Authors address:

Muluken Chanie Agimas (MC): mulukensrc12@gmail.com

Nebiyu Mekonnen Derseh (NMD): nebiyumek12@gmail.com

Fantu mamo(FM): fantuma3@gmail.com

Moges Tadesse Abebe (MTA): moges7045@gmail.com

Tilahun Yemanu(TY): yemanu.tilahun@gmail.com

Meron Asmamaw(MA): merryalem101@gmail.com

Corresponding Author

MC: mulukensrc12@gmail.com

To: PLOS ONE <em@editorialmanager.com>

Manuscript Number: PONE-D-24-00667

<u>Cover Letter for reviewer's comments and authors responses</u>

We would like to thank both the Editorial board members and reviewers for kind comments and suggestions. Accordingly, we have tried to correct and respond to those comments point by point, page by page, and line by line. We accept all the editorial errors and corrected accordingly. For those issues needs clarifications, we prepared this author's response, the revised manuscript, the separate revised figures and tables, and the tracked changes of the manuscript.

Sincerely, with best regards!

Muluken Chanie Agimas

ID: https://orcid.org/0000-0003-0808-124X

Comments from review and editorial board	Authors response
**Reviewers comment**	
Shorten the abstract to about 300 words. Merge the objective to background in the abstract section	Dear reviewer Thank you so much for your detail and excellent view, and we are lucky to have you review my research. We took a lesson from your view and your constructive comments. Therefore amendment has made accordingly.
Abstracts<br><br>In the result section, it Where is the treated. Three outcomes you should write here , burden , trend and factors	Sorry dear reviewer for missing the third objective. Now correction has made accordingly.
Abstracts<br>In the conclusion part, Do not repeat the result part here. you can summarize these one. You can say “individual level variables were identified that	Dear reviewer, we accept the comment.

affect the undiagnosed hypertension". Trend also important to mention here.	
In the searching strategy please write their name in Abbreviations	Dear reviewer, We accepted the comment, and we revised accordingly.
Table 1 should be submitted as supplementary	Dear reviewer Thank you so much for your excellent view. An amendment has been made.
In the study selection and quality appraisal, Please change this phrase, this vague. This not clear whether you planned to included only low risk. Make it clear	That is great view. We accept the comment and amendment has made accordingly.
How do you manage if the different studies used different cut point for HPN diagnosis? Is all used the same cut point?	That is great view. But we did not find out articles in which define/measure hypertension differently.
Are studies community based or included institutional a also?	Dear reviewer, we included both community based and institutional based studies.

Why prospective court only? why nor retrospective	Dear reviewer, retrospective studies were also consider and a necessary amendment has been made accordingly.
What inter sectional study mean?	Dear reviewer, it was an editorial error. Rather it mean Crossectional study.
**in the outcome of the study:**<br><br>Is the trend not your outcome?	Dear reviewer thank you so much for your suggestion. The trend of undiagnosed hypertension was our objective and thus correction has made accordingly.
The majority of the study were from Ethiopia, be sure that there was no researching bias.	Dear reviewer/editor: you are right majority of the study was from Ethiopia. We assure that we search the articles exhaustively, carefully and comprehensively one by one but we did not find out as much as in Ethiopia. Thus there is no missed article in the specified countries.

In table 2<br><br>The tittle is not complete, Pease add, undiagnosed HPN Included, the study setting in the table, community or institutional	Dear reviewer/editor, we accept and correction has made accordingly.
Only one study was included from Eretria. You included it in the subgroup analysis. For subgroup at least two study should be included in each group. Please remove the Eretria from subgroup	Dear reviewer, an amendment has been made accordingly.
Is this change significant , please indicate	Dear reviewer, the trend was statistically significant. We corrected it accordingly.
What dose this mean? Is it supplementary? where is S-1. S-2 included many figures group as S-2. Please label each figure as s-1, s-2…... and special in each main text accordingly. Apply for the rest below	Dear reviewer, an amendment has been made accordingly.
What are the limitations? specially related to the hetrogenicity ?, representaiveness in the horn of Africa ?..................., definition of Cutpoint for BP	Dear reviewer it is great. The issues what you have mentioned are the limitations of the study and therefore amendment has

categorization,	made accordingly. Regarding the cut of point, articles define and measure the blood pressure similarly.

Thank you, dear editor and reviewers, for your great scientific contribution!
