# Supplementary material for: Trend, burden and determinants of undiagnosed hypertension in the Horn of Africa: A systematic review and meta-analysis
Source: PLoS One. 2024 Aug 23;19(8):e0303940. doi: 10.1371/journal.pone.0303940 (PMC11343413; doi:10.1371/journal.pone.0303940)
Supplement: Attachment — Submitted filename: Comments and questions.docx [file pone.0303940.s006.docx]

**Comments and questions**

Firstly, I want to express my gratitude for inviting me to review this article. I have reviewed the paper and have provided specific comments and questions as follows:

In general, your study appears to be well-conducted. However, I have a major concern regarding the risk factors you identified for undiagnosed hypertension. You have listed distance from the health facility, regular exercise, age, sex, knowledge, BMI, health seeking behavior, low vegetable consumption, smoking, high triglyceride levels (TGL), chat chewing, and alcohol consumption as risk factors for the higher prevalence of undiagnosed hypertension. While it is true that factors such as distance from the health facility, age, sex, knowledge, BMI, and health seeking behavior can contribute to both the decrease or increase of undiagnosed rates and the prevalence of hypertension, I have a question regarding the role of regular exercise, low vegetable consumption, smoking, high TGL, chat chewing, and alcohol consumption in causing the higher prevalence of undiagnosed hypertension. Although these factors are known risk factors for the development of hypertension, they may not directly cause a person to remain undiagnosed. Therefore, it might be necessary to revise the factors that specifically contribute to the occurrence of undiagnosed hypertension, given that one of your research objectives is to identify the factors influencing the prevalence of undiagnosed hypertension.

It is possible that there are other determining factors, such as cultural practices or beliefs, socioeconomic factors etc. in addition to the ones you mentioned, that contribute to the undiagnosed status.

**Specific comments and comments**

**Title page:**

1. It might be better to include "horn of Africa" among your key words rather than **"Ethiopia."**

**Abstract section:**

1. You have to make it I^2^ not I2.
2. How may the less than 35-minute journey time to a medical facility lead to an underdiagnosis of hypertension? Kindly give this some further thought. in particular, the symbol "<". Rewriting it to read as follows would make it clearer: “a trip time to a medical institution of more than 35 minutes may be a factor in the high rate of undiagnosed hypertension.”
3. In your conclusion section you said, less than 35 minutes of travel time was found to be protective against undiagnosed hypertension. Do you believe that in the case of undiagnosed hypertension, it is reasonable to say protective unprotective? Being undiagnosed itself is a problem. I believe it would be more acceptable to say either raise or decrease the rate of undiagnosed.

**Introduction section:**

1. Nine million ischemic heart disease and 3.5 million strokes can be attributed to high blood pressure. lines 77–78 on page 4. Is it every year? Please explain. Additionally, ischemic should be used instead of ischaemic.
2. Space out the word **Anddeterminats** on page 5, line 96.

**Methodology section:**

**Design:**

1. Describe your study design here first, and then you can mention as you used the PRISMA-P 2015 Guidelines to report it not to design.

**Searcing strategy:**

1. Make it searching not searcing.
2. Using the search terms like "undetected hypertension, systolic blood pressure and/or diastolic blood pressure" may have increased the quantity of papers you found for review.

**Data analysis:**

1. Please remove the word “anal” on page 8, line 161.

**Result section:**

1. What does it mean a **crossconditional** study design? On page 9, line 177.
2. Please spaced out it “Egger’sstatistical” on page 9, 190
3. Give space for “factor(Table-3, S-2).” On page 11, line 234.
4. Make subscript the word “was” **there ^was^ no**. on page 12, line 248
